# Complex genetic variation in nearly complete human genomes

**DOI:** 10.1038/s41586-025-09140-6

**Published:** 2025-07-23

**Authors:** Glennis A. Logsdon, Peter Ebert, Peter A. Audano, Mark Loftus, David Porubsky, Jana Ebler, Feyza Yilmaz, Pille Hallast, Timofey Prodanov, DongAhn Yoo, Carolyn A. Paisie, William T. Harvey, Xuefang Zhao, Gianni V. Martino, Mir Henglin, Katherine M. Munson, Keon Rabbani, Chen-Shan Chin, Bida Gu, Hufsah Ashraf, Stephan Scholz, Olanrewaju Austine-Orimoloye, Parithi Balachandran, Marc Jan Bonder, Haoyu Cheng, Zechen Chong, Jonathan Crabtree, Mark Gerstein, Lisbeth A. Guethlein, Patrick Hasenfeld, Glenn Hickey, Kendra Hoekzema, Sarah E. Hunt, Matthew Jensen, Yunzhe Jiang, Sergey Koren, Youngjun Kwon, Chong Li, Heng Li, Jiaqi Li, Paul J. Norman, Keisuke K. Oshima, Benedict Paten, Adam M. Phillippy, Nicholas R. Pollock, Tobias Rausch, Mikko Rautiainen, Yuwei Song, Arda Söylev, Arvis Sulovari, Likhitha Surapaneni, Vasiliki Tsapalou, Weichen Zhou, Ying Zhou, Qihui Zhu, Michael C. Zody, Ryan E. Mills, Scott E. Devine, Xinghua Shi, Michael E. Talkowski, Mark J. P. Chaisson, Alexander T. Dilthey, Miriam K. Konkel, Jan O. Korbel, Charles Lee, Christine R. Beck, Evan E. Eichler, Tobias Marschall

**Affiliations:** 1 Department of Genome Sciences, University of Washington School of Medicine, Seattle, WA USA; 2 Department of Genetics, Epigenetics Institute, Perelman School of Medicine, University of Pennsylvania, Philadelphia, PA USA; 3 Core Unit Bioinformatics, Medical Faculty and University Hospital Düsseldorf, Heinrich Heine University, Düsseldorf, Germany; 4 Center for Digital Medicine, Heinrich Heine University, Düsseldorf, Germany; 5 The Jackson Laboratory for Genomic Medicine, Farmington, CT USA; 6 Department of Genetics and Biochemistry, Clemson University, Clemson, SC USA; 7 Center for Human Genetics, Clemson University, Greenwood, SC USA; 8 Institute for Medical Biometry and Bioinformatics, Medical Faculty and University Hospital Düsseldorf, Heinrich Heine University, Düsseldorf, Germany; 9 Program in Medical and Population Genetics and Stanley Center for Psychiatric Research, Broad Institute of MIT and Harvard, Cambridge, MA USA; 10 Center for Genomic Medicine, Massachusetts General Hospital, Boston, MA USA; 11 Department of Neurology, Massachusetts General Hospital and Harvard Medical School, Boston, MA USA; 12 Medical University of South Carolina, College of Graduate Studies, Charleston, SC USA; 13 Department of Quantitative and Computational Biology, University of Southern California, Los Angeles, CA USA; 14 Pathos AI Inc., Chicago, IL USA; 15 Institute of Medical Microbiology and Hospital Hygiene, Medical Faculty, Heinrich Heine University, Düsseldorf, Germany; 16 European Molecular Biology Laboratory, Wellcome Genome Campus, European Bioinformatics Institute, Cambridge, UK; 17 Department of Genetics, University of Groningen, University Medical Center Groningen, Groningen, The Netherlands; 18 Oncode Institute, Utrecht, The Netherlands; 19 Division of Computational Genomics and Systems Genetics, German Cancer Research Center, Heidelberg, Germany; 20 Department of Biomedical Informatics and Data Science, Yale School of Medicine, New Haven, CT USA; 21 Department of Biomedical Informatics and Data Science, Heersink School of Medicine, University of Alabama, Birmingham, AL USA; 22 Institute for Genome Sciences, University of Maryland School of Medicine, Baltimore, MD USA; 23 Department of Molecular Biophysics and Biochemistry, Yale University, New Haven, CT USA; 24 Program in Computational Biology and Bioinformatics, Yale University, New Haven, CT USA; 25 Department of Structural Biology, School of Medicine, Stanford University, Stanford, CA USA; 26 Genome Biology Unit, European Molecular Biology Laboratory (EMBL), Heidelberg, Germany; 27 UC Santa Cruz Genomics Institute, University of California, Santa Cruz, CA USA; 28 Genome Informatics Section, Center for Genomics and Data Science Research, National Human Genome Research Institute, National Institutes of Health, Bethesda, MD USA; 29 Department of Computer and Information Sciences, College of Science and Technology, Temple University, Philadelphia, PA USA; 30 Institute for Genomics and Evolutionary Medicine, Temple University, Philadelphia, PA USA; 31 Department of Data Science, Dana-Farber Cancer Institute, Boston, MA USA; 32 Department of Biomedical Informatics, Harvard Medical School, Boston, MA USA; 33 Department of Biomedical Informatics, University of Colorado School of Medicine, Aurora, CO USA; 34 Department of Immunology and Microbiology, University of Colorado School of Medicine, Aurora, CO USA; 35 Institute for Molecular Medicine Finland (FIMM), University of Helsinki, Helsinki, Finland; 36 Department of Computational Medicine and Bioinformatics, University of Michigan, Ann Arbor, MI USA; 37 Stanford Health Care, Palo Alto, CA USA; 38 New York Genome Center, New York, NY USA; 39 The University of Connecticut Health Center, Farmington, CT USA; 40 Howard Hughes Medical Institute, University of Washington, Seattle, WA USA; 41 Present Address: The Jackson Laboratory for Genomic Medicine, Farmington, CT USA

**Keywords:** Genomics, Genome informatics, Immunogenetics, Structural variation

## Abstract

Diverse sets of complete human genomes are required to construct a pangenome reference and to understand the extent of complex structural variation. Here we sequence 65 diverse human genomes and build 130 haplotype-resolved assemblies (median continuity of 130 Mb), closing 92% of all previous assembly gaps^1,2^ and reaching telomere-to-telomere status for 39% of the chromosomes. We highlight complete sequence continuity of complex loci, including the major histocompatibility complex (MHC), *SMN1*/*SMN2*, *NBPF8* and *AMY1/AMY2*, and fully resolve 1,852 complex structural variants. In addition, we completely assemble and validate 1,246 human centromeres. We find up to 30-fold variation in α-satellite higher-order repeat array length and characterize the pattern of mobile element insertions into α-satellite higher-order repeat arrays. Although most centromeres predict a single site of kinetochore attachment, epigenetic analysis suggests the presence of two hypomethylated regions for 7% of centromeres. Combining our data with the draft pangenome reference^1^ significantly enhances genotyping accuracy from short-read data, enabling whole-genome inference^3^ to a median quality value of 45. Using this approach, 26,115 structural variants per individual are detected, substantially increasing the number of structural variants now amenable to downstream disease association studies.

## Abstract (web summary)

Using sequencing and haplotype-resolved assembly of 65 diverse human genomes, complex regions including the major histocompatibility complex and centromeres are analysed.

## Main

Long-read sequencing (LRS) technologies were critical to the completion of the first human genome^4^. LRS technologies significantly increase the sensitivity to detect structural variants (SVs), defined as variants 50 bp in length or longer, and coupling LRS data with Hi-C^5^, single-cell template strand sequencing (Strand-seq)^6^ or trio data^7^ provided the necessary short-range and long-range phasing data to assemble both haplotypes. The high sequence quality and contiguity of such diploid genome assemblies have made the first draft human pangenome reference possible^1^.

Despite these advances, gaps remain, especially at genetically complex loci^2^. For example, in our previous assembly of 32 human genomes as part of the Human Genome Structural Variation Consortium (HGSVC)^8^, we found that most centromeres and more than half of the large, highly identical segmental duplications (SDs) were incomplete, resulting in missing protein-coding genes^2^. Closing these gaps in the first complete human genome^4^ required combining the complementary strengths of PacBio high-fidelity (HiFi) reads (approximately 18 kb in length and high base-level accuracy) and ultra-long Oxford Nanopore Technologies (ONT) reads (more than 100 kb in length but with lower base-level accuracy). Computational tools such as Verkko^9^ and hifiasm (ultra-long)^10^ have automated this process. Here we present new resources and results from the HGSVC (Supplementary Fig. 1), targeting a diverse set of 65 humans predominantly from the 1000 Genomes Project (1kGP) cohort^11^ with the goal of producing a genetically diverse sampling of nearly gapless chromosomes, including the centromeres and complex SDs.

## Production of 130 haplotype assemblies

### Data production

**Fig. 1:**
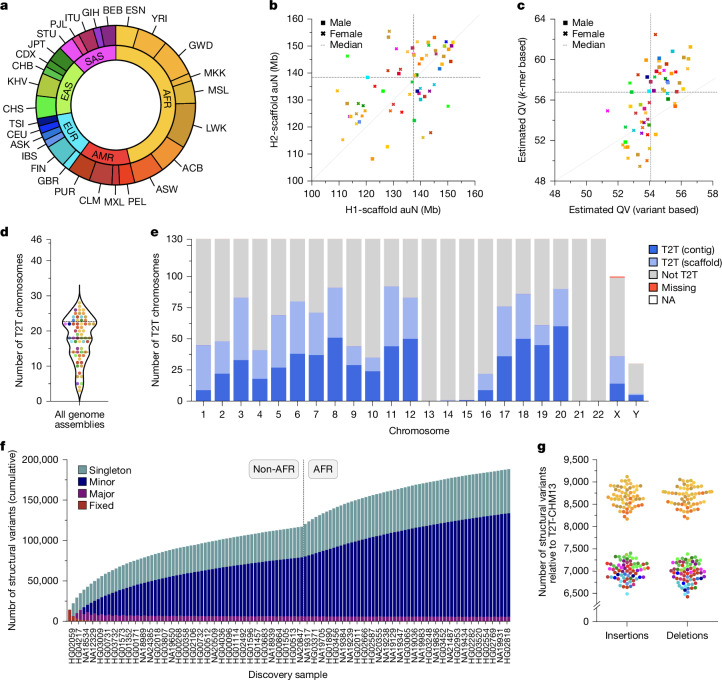
LRS, assembly and variant calling of 65 diverse humans. **a**, Continental group (inner ring) and population group (outer ring) of the 65 diverse humans analysed in this study. AFR, African; AMR, American; EAS, East Asian; EUR, European; SAS, South Asian. Population groups are labelled according to the 1000 Genomes Project^11^, along with the added Maasai in Kinyawa, Kenya (MKK) and Ashkenazim (ASK) labels. **b**, Scaffold auN for haplotype 1 (H1) and haplotype 2 (H2) contigs from each genome assembly. Data points are coloured by population group. The dashed lines indicate the median auN per haplotype. The dotted line indicates the unit diagonal. **c**, Quality value (QV) estimates for each genome assembly derived from variant calls or *k*-mer statistics (Methods). **d**, The number of chromosomes assembled from T2T for each genome assembly, including both single contigs and scaffolds (Methods). The median (solid line) and first and third quartiles (dotted lines) are shown. **e**, The number of T2T chromosomes in a single contig (dark blue, T2T contig) or in a single scaffold (light blue, T2T scaffold). Incomplete chromosomes are labelled as ‘not T2T’ or ‘missing’ if missing entirely. Sex chromosomes not present in the respective haploid assembly are labelled as ‘NA’. **f**, Cumulative non-redundant SVs across the diverse haplotypes in this study called with respect to the T2T-CHM13 reference genome (three trio children excluded). **g**, Number of SVs detected for each haplotype relative to the T2T-CHM13 reference genome, coloured by population. Insertions and deletions are balanced when called against the T2T-CHM13 reference genome but imbalanced when called against the GRCh38 reference genome (Extended Data Fig. 1d).

**Extended Data Fig. 1:**
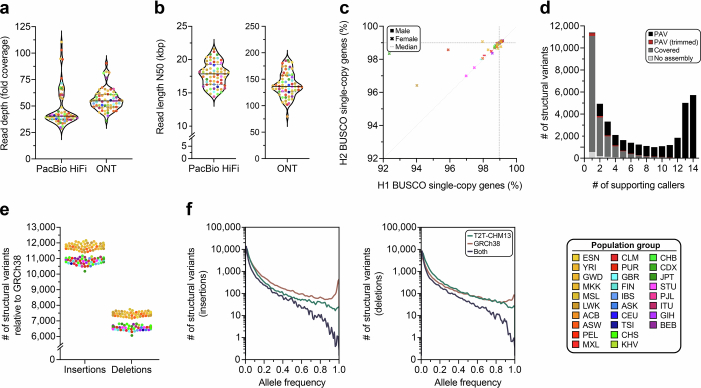
Statistics of long-read sequencing data and genome assemblies generated in this study as well as variant calls for 65 diverse human genomes. **a**) Fold coverage of the Pacific Biosciences (PacBio) high-fidelity (HiFi) and Oxford Nanopore Technologies (ONT) long-read sequencing data generated for each genome in this study. The median (solid line) and first and third quartiles (dotted lines) are shown. **b**) Read length N50 of the PacBio HiFi and ONT data generated for each genome in this study. The median (solid line) and first and third quartiles (dotted lines) are shown. **c**) Gene completeness as a percentage of BUSCO single-copy orthologs detected in each haplotype from each genome assembly (Methods). **d**) The number of SVs identified in one individual by 14 different SV callers, including PAV (Methods). Each bar is divided into four categories as follows: PAV, SVs identified by PAV (black); PAV (trimmed), false SVs from other callers in redundantly aligned sequences that PAV removes (red); Covered, SVs not called by PAV but within callable loci spanned by assembly alignments (dark gray); No assembly, SVs identified in locations not callable by PAV (light gray). Before applying caller-based QC, 99.75% of PAV calls are supported by at least one other call source. The individual evaluated is HG00171. **e**) Number of SVs called for each haplotype relative to the GRCh38 reference genome, colored by population. Insertions and deletions are imbalanced when called against the GRCh38 reference genome but balanced when called against the T2T-CHM13 reference genome (Fig. 1g). **f**) Number of SV insertions (left) and deletions (right) called against T2T-CHM13, GRCh38, or both reference genomes relative to their allele frequency. SVs called against both references tend to be rarer because they are less likely to appear in a reference genome. A sharp peak for high allele frequency (~1.0) for insertions is detected relative to the GRCh38 reference genome but not the T2T-CHM13 reference genome.

We selected 65 human lymphoblastoid cell lines representing individuals spanning five continental groups and 28 population groups for sequencing (Fig. 1a and Supplementary Table 1). We generated approximately 47-fold coverage of PacBio HiFi and approximately 56-fold coverage of ONT (approximately 36-fold ultra-long) long reads on average per individual (Extended Data Fig. 1a,b and Supplementary Table 2; see Methods). In addition, we performed Strand-seq (Supplementary Table 2), Bionano Genomics optical mapping (Supplementary Table 3), Hi-C sequencing (Supplementary Tables 4 and 5), isoform sequencing (Iso-Seq; Supplementary Table 6) and RNA sequencing (RNA-seq; Supplementary Table 7).

### Assembly

We generated haplotype-resolved assemblies from all 65 diploid individuals using Verkko^9^ (Fig. 1a and Supplementary Tables 1 and 2; see Methods). The phasing signal was produced with Graphasing^12^, leveraging Strand-seq to globally phase assembly graphs at a quality on par with trio-based workflows^12^ (Methods). This approach enabled us to cover all 26 populations from the 1kGP by including individuals that are not part of a family trio. The resulting set of 130 haploid assemblies is highly contiguous (median area under the Nx curve (auN) of 137 Mb; Fig. 1b and Supplementary Table 8) and accurate at the base-pair level (median quality value between 54 and 57; Fig. 1c and Supplementary Table 9; see Methods). We estimated the assemblies to be 99% complete (median) for known single-copy genes (Extended Data Fig. 1c and Supplementary Table 10) and to close 92% of previously reported gaps in PacBio HiFi-only assemblies^2^ (Supplementary Figs. 2 and 3 and Supplementary Table 11; see Methods).

We integrated a range of quality control annotations for each assembly using established tools such as Flagger, NucFreq, Merqury and Inspector (Supplementary Tables 12 and 13 and Figs. 4 and 5) to compute robust error estimates for each assembled base (Supplementary Tables 14–17; see Methods). We estimated that 99.6% of the phased sequence (median) has been assembled correctly (Supplementary Table 18). For the three family trios in our dataset (SH032, Y117 and PR05 (ref. ^11^)), we assessed the parental support for the respective haplotypes in the child’s assembly via assembly-to-assembly alignments and found that a median of 99.9% of all sequence assembled in contigs of more than 100 kb are supported by one parent assembly (Supplementary Table 19; see Methods). In total, Verkko assembled 602 chromosomes as a single gapless contig from telomere to telomere (T2T; median of 10 per genome) and an additional 559 as a single scaffold (median of 8 per genome), that is, in a connected sequence containing one or more N-gaps (Fig. 1d,e, Supplementary Table 20 and Supplementary Fig. 6; see Methods).

Certain regions, such as centromeres or the Yq12 region, remained challenging to assemble and evaluate. We therefore complemented our assembly efforts by running hifiasm (ultra-long)^10^ on the same dataset (Supplementary Tables 21–23 and Supplementary Figs. 7 and 8; see Methods), but restricted the use of the resulting assemblies to extending our analysis set for centromeres and the Yq12 region after manual curation of the relevant sequences.

### Variant calling

From our phased assemblies, we identified 188,500 SVs, 6.3 million indels and 23.9 million single-nucleotide variants (SNVs) against the T2T-CHM13v.2.0 (T2T-CHM13) reference (Fig. 1f). Against GRCh38-NoALT (GRCh38), we identified 176,531 SVs, 6.2 million indels and 23.5 million SNVs (Supplementary Table 24; see Data availability). Callsets for both references were led by PAV (v.2.4.0.1)^8^ with orthogonal support from 10 other independent callers with sensitivity for SVs, indels and SNVs (Supplementary Table 25; see Methods). We found higher support for PAV calls across all callers (99.7%) than other methods (99.7% to 67.9%; Extended Data Fig. 1d and Supplementary Fig. 9), with one exception for SVIM-asm, when run using the alignment parameters for PAV (99.70% SVIM-asm versus 99.66% PAV; Supplementary Table 26). With our current assemblies and this approach, we increased the size of the SV callset by 59% and reduced false discovery by 55% on average compared with previous callsets^8^ (Supplementary Tables 27 and 28 and Supplementary Methods). With one additional individual, we estimated that our callset would increase by 842 SV insertions and deletions with a 1.86× enrichment for an African versus a non-African individual (1,117 versus 599; Supplementary Methods).

Per assembled haplotype, we identified 7,772 SV insertions (12,903 per genome) and 7,745 SV deletions (12,505 per genome) on average in the T2T-CHM13 reference (Fig. 1g). As expected, GRCh38 SVs are unbalanced^8,13^ with 11,275 SV insertions per haplotype (17,458 per genome) and 6,972 SV deletions per haplotype (10,868 per genome) on average (Extended Data Fig. 1e and Supplementary Tables 29 and 30), with excess insertions occurring in high-allele-frequency variants, which can be largely explained by reference errors^14^. As expected, a distinct peak for fixed SVs (100% allele frequency) is apparent for GRCh38 SV insertions composed of variants in GRCh38 with no representation in T2T-CHM13 (Extended Data Fig. 1f).

## An improved genomic resource

### Mobile element insertions

Mobile element insertions (MEIs)^15^ constitute 8.2% of all SVs (relative to T2T-CHM13). We identified 12,919 putative MEIs from the 130 haplotype assemblies (Supplementary Table 31 and Supplementary Fig. 10; see Methods; for the GRCh38 union callset, see Supplementary Table 32 and Supplementary Fig. 11). Comparison with an orthogonal MEI callset showed a high concordance of 92.1% (Supplementary Tables 33 and 34; see Methods). Of note, we found 559 full-length L1 insertions (L1HS and L1PA2), with 96.1% possessing at least one intact open reading frame (ORF) and 82.3% harbouring two intact ORFs. Therefore, the vast majority of full-length L1 MEIs appear to retain the potential to retrotranspose. Compared with our previous study^8^ (*n* = 9,453 MEIs; 7,738 for *Alu*, 1,775 for L1 and 540 for SINE-VNTR-Alu (SVA)), the total number of MEIs increased by 36.65% primarily due to an increase in individuals of African descent (Supplementary Fig. 10d). Finally, we screened the PAV deletion callset and identified 2,450 polymorphic MEIs present in T2T-CHM13 (Supplementary Tables 35 and 36 and Supplementary Fig. 12).

### Inversions

Identifying inversions is challenging due to the frequent location of their boundaries in long, highly identical repeat sequences. We identified 276 T2T-CHM13-based and 298 GRCh38-based inversions in the main callset and performed quality control by re-genotyping these calls using ArbiGent on Strand-seq data^16^ (Supplementary Tables 37 and 38 and Supplementary Methods) as well as manual inspection (Supplementary Table 37, Supplementary Figs. 13 and 14 and Supplementary Methods). Of note, we found 21 novel inversions in the PAV callset, of which 18 were detected among 24 new individuals added in the current study. These include a large (1.8 Mb) inversion at chromosome 5q35 that overlaps with the Sotos syndrome critical region^17^.

### Segmental duplications

**Extended Data Fig. 2:**
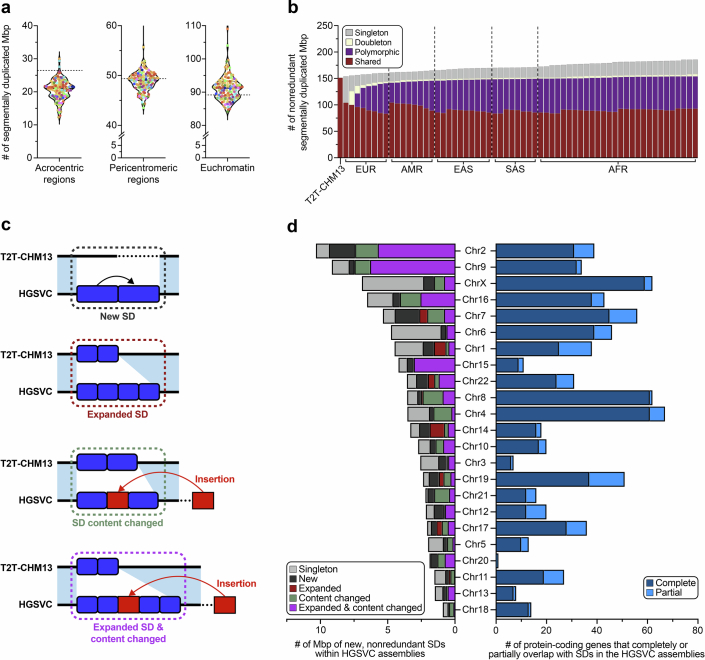
Classification and distribution of changes in SD content in the 65 genomes. **a**) Number of segmentally duplicated bases assembled in different regions of the genome for each individual in this study, excluding sex chromosomes. The dashed line indicates the number of segmentally duplicated bases in the T2T-CHM13 genome. **b**) Segmental duplication (SD) accumulation curve. Starting with T2T-CHM13, the SDs (excluding those located in acrocentric regions and chrY) of 63 individuals (excluding NA19650 and NA19434) were projected onto T2T-CHM13 genome space in the continental group order of: EUR, AMR, EAS, SAS and AFR. For each bar, the SDs that are singleton, doubleton, polymorphic (>2) and shared (>90%) are indicated. The first bar is classified as “shared”, as the assembly is only being compared to itself. **c**) Schematic depicting the four categories of non-reference SDs: 1) new (i.e., unique in the reference), 2) expanded copy number, 3) content or composition changed, and 4) expanded and content changed SDs with respect to the SDs in the reference genome, T2T-CHM13. **d**) Quantification in terms of Mbp and predicted protein-coding genes across the four categories of new SDs compared to T2T-CHM13. The left panel shows the Mbp by category, while flagging those that are singleton (i.e., duplicated in T2T-CHM13 but not in other genomes). The right panel quantifies the number of complete (100% coverage) and partial overlaps (>50% coverage) with protein-coding genes for the respective chromosomes.

SDs are defined independently for each haplotype as segments occuring more than once with more than 1 kb in length and more than 90% identity. Owing to their propensity to undergo non-allelic homologous recombination, they are enriched tenfold for copy number variation and are the source of some of the most complex forms of genic structural polymorphism in the human genome^18,19^. Overall, we found an average of 168.1 Mb (s.d. of 9.2 Mb) of SDs per human genome and observed an improved representation of interchromosomal SDs (Supplementary Figs. 15 and 16) when compared with the Human Pangenome Reference Consortium (HPRC) release^1^. Using T2T-CHM13 as a gauge of completeness (193.7 Mb), we estimated that 25.6 Mb of SDs still remain unresolved per haploid genome (Extended Data Fig. 2a). Most of these unresolved SDs (21.2 Mb) correspond to the acrocentric short arms of chromosomes 13, 14, 15, 21 and 22 (refs. ^4,20^). We found that 80–90% of SDs are accurately assembled depending on the genome (Supplementary Figs. 17 and 18; see Methods).

When analysing SDs outside of acrocentric regions and where the copy number was supported by fastCN (Supplementary Fig. 19; see Methods), we classified at least 92.8 Mb of the SDs as shared among most humans (present in at least 90% of individuals) and 61.0 Mb as variable across the human population (Extended Data Fig. 2b). In addition, we identified 33 Mb of the SD sequence present in a single copy or not annotated as SDs in T2T-CHM13 (Extended Data Fig. 2c,d). The majority of these (23.6 Mb, including 2.4 Mb of X chromosome SDs) are novel when compared with a recent analysis of 170 human genomes^21^ and completely or partially overlap with 167 protein-coding genes (Supplementary Fig. 20). Of note, 31 loci (0.4 Mb) are shared among most humans but not classified as duplicated in the T2T-CHM13 human genome, suggesting that this unique status in the reference represents the minor allele in the human population, a cell line artefact or, less likely, an error in the assembly. Examining genomes by continental group, both the absolute SD content^21^ (Supplementary Figs. 21 and 22) and the number of new SDs added per genome is highest for African individuals (3.97 Mb per individual) when compared with genomes of non-African individuals (2.88 Mb per individual).

Genomes with African ancestry have, on average, 468 additional paralogous genes (*n* = 21,595 total genes) when compared with genomes of non-African individuals (*n* = 21,127 total genes; Methods). We identified a total of 727 multi-copy genes that have SDs spanning at least 90% of the gene body, with a large proportion corresponding to shared (*n* = 335 or 46.1%) and variable (*n* = 292 or 40.2%) SDs (Supplementary Table 39). Comparing the copy numbers to the HPRC assemblies^1^, we discovered a similar distribution of genes (Supplementary Fig. 23). Among copy number polymorphic genes, we identified 16 gene families in which the distribution significantly differs between the HPRC and our data (Supplementary Fig. 23; adjusted *P* < 0.05, two-sided Welch’s *t*-test); however, the contiguity for copy number variant genes was considerably greater in our assemblies versus HPRC; 5.88% of duplicated genes in our assemblies are within 200 kb of a contig break or unknown base (‘N’) compared with 13.95% of duplicated genes in HPRC assemblies (Supplementary Fig. 24).

### Y chromosome variation

**Fig. 2:**
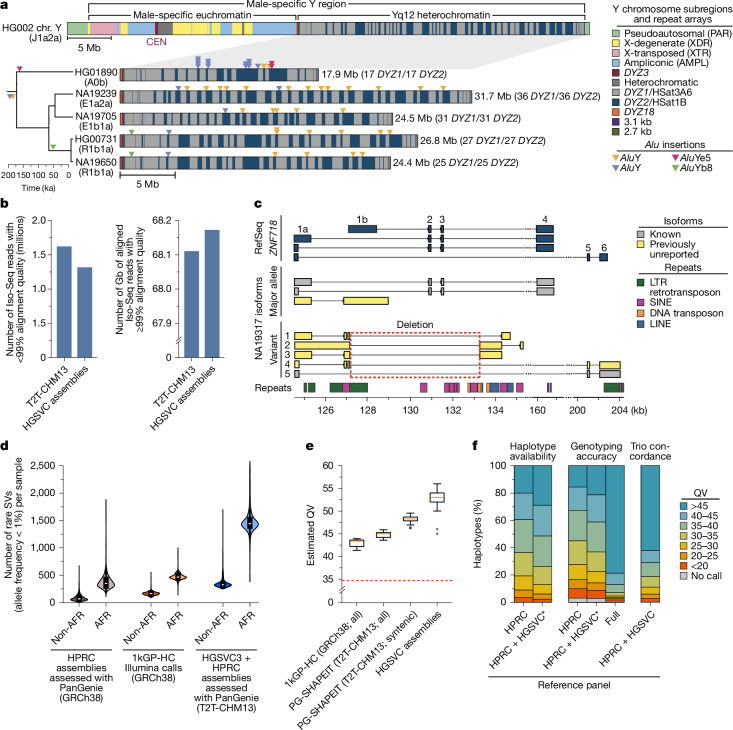
An improved genomic resource for challenging loci. **a**, Structure of a human Y chromosome, including the centromere (CEN; top), and repeat composition of five contiguously assembled Yq12 heterochromatic regions with their phylogenetic relationships (bottom left), size or number of *DYZ1* and *DYZ2* repeat array blocks (bottom right), and *Alu* insertion locations (triangles). ka, thousand years ago. **b**, Number of Iso-Seq reads that fail to align with 99% or less accuracy (left), and number of gigabases (Gb) of Iso-Seq reads that align with 99% or more accuracy (right) to the T2T-CHM13 reference genome versus the assemblies in this study. **c**, Expressed isoforms of *ZNF718* in NA19317. This individual is heterozygous for a deletion (red box, chr. 4: 127125–133267) that affects the *ZNF718* exon–intron structure. Isoforms not previously annotated in RefSeq, GENCODE or CHESS (Methods) are shown (yellow). LTR, long terminal repeat; SINE, short interspersed nuclear element; LINE, long interspersed nuclear element. **d**, Number of rare (allele frequency < 1%) SVs per sample in the HPRC-genotyped callset (grey), Illumina-based 1kGP-HC SV callset (orange), and combined HPRC and HGSVC-genotyped callset (blue) for both non-African (non-AFR) and African (AFR) individuals (*n* = 3,202). The first and third quartiles (Q1 and Q3, respectively; black boxes), median (white dots), and minima and maxima (black lines) are shown. **e**, Estimated *k*-mer-based QV for 60 haplotypes from the 1kGP-HC-phased set (GRCh38 based), HGSVC-phased genotypes using PanGenie, SHAPEIT5 (PG-SHAPEIT, T2T-CHM13 based) and all HGSVC genome assemblies. ‘Syntenic’ refers to regions of T2T-CHM13 also present in GRCh38. Baseline QV estimated by randomizing samples (red dashed line), first and third quartiles (black boxes), median (orange line), outliers (white dots) and whiskers (quantile 1 − 1.5(quantile 3–quantile 1) and quantile 3 + 1.5(quantile 3–quantile 1)) are shown. **f**, Haplotype availability, Locityper genotyping accuracy and trio concordance across 347 polymorphic loci in terms of variant-based QV. Availability and accuracy are calculated for 61 HGSVC individuals, whereas trio concordance is calculated for 602 trios. Full, HPRC + HGSVC; HPRC, HPRC only; HPRC + HGSVC*, HPRC + HGSVC leave-one-out.

The Y chromosome remains among the most challenging of human chromosomes to fully assemble due to its highly repetitive sequence composition^20^ (Fig. 2a). Our resource provides highly contiguous Y assemblies for 30 male individuals. Seven of these (23%) assembled without breaks across the male-specific Y region (excluding the pseudoautosomal regions, six assembled as T2T scaffolds and one that has a break in the pseudoautosomal region 1; Supplementary Figs. 25 and 26). Of these seven, four are novel fully assembled human Y chromosomes representing E1b1a, R2a and R1b1a Y lineages prevalent in populations of African, Asian and European descent^22–24^ (Supplementary Fig. 27).

Our assemblies enable the investigation of the largest heterochromatic region in the human genome, Yq12, mostly composed of highly similar (but size variable) alternating arrays of *DYZ1* (*HSat3A6*, approximately 3.5-kb unit size) and *DYZ2* (*HSat1B*, approximately 2.4-kb unit size) repeats (Fig. 2a). The Yq12 regions across 16 individuals (9 novel and 7 previously published) range from 17.85 to 37.39 Mb (mean of 27.25 Mb, median of 25.62 Mb), with high levels of variation in the number (34–86 arrays; mean of 60, median of 58) and length of *DYZ1* (24.4 kb to 3.59 Mb; mean of 525.7 kb, median of 455.0 kb) and *DYZ2* (11.2 kb to 2.20 Mb; mean of 358.0 kb, median of 273.3 kb) repeat arrays^23,24^ (Supplementary Table 40 and Supplementary Fig. 28). Investigating the dynamics of Yq12 remains challenging^25^; however, using the duplication and deletion patterns of four unique *Alu* insertions, we can examine this genomic region over time (Fig. 2a and Supplementary Fig. 28). For example, in NA19239, the presence of two unique *Alu*Y retrotransposon insertions allows clear visualization of a tandem duplication in the region.

### Functional effects of SVs

To identify SVs disrupting protein-coding genes under selective constraint^26^, we intersected all 176,531 GRCh38-based SVs with coding exons from GENCODE v.45. We found 1,535 SVs, including 938 deletions, 80 inversions, 504 insertions and 13 MEIs, that disrupt 985 unique genes (Supplementary Table 41). A mean of 368 genes per genome have an SV breakpoint altering the coding sequence. On average, only 11.7 genes (3.2%) were disrupted by a singleton variant unique to that individual, whereas 96.8% of genes were disrupted by polymorphic SVs, and 27.8% were disrupted by major-allele SVs (more than 50% allele frequency). Of the 1,535 genes affected by SVs, only 37 were predicted to be intolerant to loss of function in humans (loss-of-function observed/expected upper bound fraction (LOEUF) < 0.35)^27^. Polymorphic SVs altered 16 constrained genes, suggesting that the SVs did not result in loss of function. Indeed, we found that tandem repeat unit variants in coding sequences of four constrained genes were in frame (*MUC5B*, *ACAN*, *FMN2* and *ARMCX4*). Deletion of one or more 59-bp VNTR units overlapping the last 8 bp of *MUC5B* exon 37 left coding sequences and splice sites intact.

**Extended Data Fig. 3:**
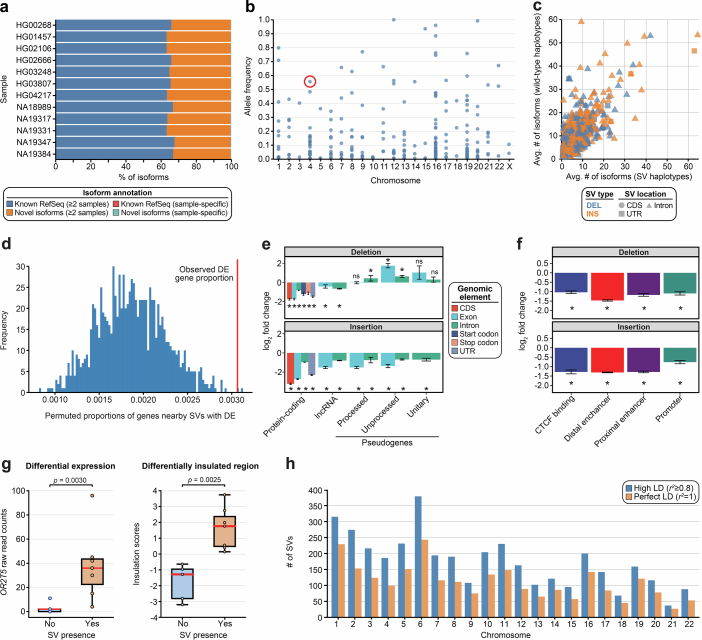
Effects of SVs on gene expression, chromosome conformation, and complex traits. **a**) The percentage of Iso-Seq isoforms identified for each individual classified as previously identified in RefSeq (present in at least two individuals; blue), novel (present in at least two individuals; orange), individual-specific previously identified isoforms (red), or individual-specific novel (teal). **b**) Manhattan plot of the allele frequencies for 256 SVs disrupting protein-coding exons of 136 genes with expression present in Iso-Seq. Circled in red is the 6,142 bp polymorphic deletion in *ZNF718*. **c**) Comparison of the average unique isoforms in Iso-Seq phased to wild-type and variant haplotypes for 1,471 single SV-containing protein-coding genes. The color represents the type of SV [deletion (DEL): blue, insertion (INS): orange] and the shape indicates where the SV occurs in relation to the canonical transcript [circle: coding sequence (CDS), square: untranslated region (UTR), triangle: intron]. **d**) Proportion of genes located within 50 kbp of SV regions that show differential expression (DE; RNA-seq) among individuals who carry the SVs (red line), compared with the distribution of DE gene proportions nearby simulated SV regions (1,000 permutations). **e**) Enrichments and depletions of SVs within GENCODE v45 protein-coding, long noncoding RNA (lncRNA), and pseudogene elements, subdivided into various biotypes. *empirical p < 0.05 from 1,000 permutations with Benjamini-Hochberg correction. ns, nonsignificant. Error bars indicate ±1 s.d. centered on the mean. p-values are listed in Supplementary Table 43. **f**) Enrichments and depletions of SVs within classes of ENCODE candidate cis-regulatory elements (cCREs). *empirical p < 0.05 from 1,000 permutations with Benjamini-Hochberg correction. ns, nonsignificant. Error bars indicate ±1 s.d. centered on the mean. p-values are listed in Supplementary Table 59. **g**) A differentially insulated region in individuals with chr1-248444872-INS-63 SV, located nearby the DE gene *OR2T5*, suggests an SV-mediated novel chromatin domain could lead to increased gene expression. n = 7 individuals with the SV and 5 without the SV. Box plots indicate median and first and third quartiles, with whiskers extending to 1.5 times the interquartile range. Two-sided Wilcoxon rank-sum test with Benjamini-Hochberg correction. **h**) Number of SVs per chromosome that are in high (r^2^ > 0.8) or perfect (r^2^ = 1) linkage disequilibrium (LD) with GWAS SNPs significantly associated with diseases and human traits.

To assess isoform differences and detect imprinted genes, we generated long-read Iso-Seq data for 12 of the 65 individuals (EBV-transformed lymphoblastoid B cell lines) and aligned these to donor-matched haplotype assemblies (Fig. 2b, Extended Data Fig. 3a and Supplementary Methods). Using our SV callset (Methods), we identified 136 structurally variable protein-coding gene sequences (Supplementary Table 42 and Supplementary Methods). Of these 136 genes, 58% (*n* = 79) contained a common SV (allele frequency > 0.05; Extended Data Fig. 3b). One example, *ZNF718*, creates nine unique isoforms (Fig. 2c) due to a common (allele frequency = 0.55) 6,142-bp polymorphic deletion that removes exons 2 and 3 from the canonical transcript as well as the 3′ part of an exon annotated as an alternate first exon (Extended Data Fig. 3b). Across the 14 wild-type *ZNF718* haplotypes, we found three known isoforms and four previously unreported isoforms (Methods). In contrast to other protein-coding genes with a single SV (Extended Data Fig. 3c), we found greater transcript diversity among the variant haplotypes of *ZNF718* than wild-type haplotypes. We also searched for SVs affecting nearby gene expression (RNA-seq) and identified 122 unique SVs proximal (less than 50 kb) to 98 differentially expressed genes across the 12 individuals, representing an enrichment compared with randomly permuted SVs (Extended Data Fig. 3d; empirical *P* = 0.001; Supplementary Table 43 and Supplementary Fig. 29; see Methods). Genome-wide, SVs were depleted across protein-coding genes and regulatory regions in the genome, as expected^28^ (Extended Data Fig. 3e,f and Supplementary Fig. 30). By intersecting these 122 SVs with Hi-C data from the same individuals, we found that 29 of the SVs (associated with 24 genes) correspond to contact density changes in chromatin conformation regions (Extended Data Fig. 3g, Supplementary Table 44 and Supplementary Methods). Finally, we identified 3,818 SVs in high linkage disequilibrium with single-nucleotide polymorphism (SNP) loci from genome-wide association studies (GWAS) of human disease (Extended Data Fig. 3h and Supplementary Table 45; see Methods).

## Genotyping and integrated reference panel

### Genome-wide genotyping with PanGenie

Pangenome references have enabled genome inference, a process leveraging haplotype structures to genotype all variation encoded within a pangenome in a new individual from short-read whole-genome sequencing data^3^. We therefore constructed a pangenome graph containing all 65 genomes assembled here as well as 42 HPRC genome assemblies^1^ with Minigraph-Cactus and detected variants by identifying graph bubbles relative to T2T-CHM13 (Methods). We used PanGenie to genotype bubbles across all 3,202 individuals from the 1kGP cohort based on Illumina data^29^ and decomposed the 30,490,169 bubbles into 28,343,728 SNPs, 10,421,787 indels and 547,663 SV alleles^1^ (Supplementary Fig. 31; see Methods). Leave-one-out experiments confirmed high genotype concordance of up to approximately 94% for biallelic SVs (Supplementary Figs. 32–34), and filtering the genotypes^1,8^ resulted in a set of reliably genotypable variants comprising 25,695,951 SNPs, 5,774,201 indels and 478,587 SV alleles (Supplementary Table 46, Supplementary Figs. 35 and 36 and Supplementary Methods). We note that this set of SV alleles is larger than our main PAV callset (188,500 SVs) because it includes the HPRC genome assemblies and at the same time retains all SV alleles at multi-allelic sites (Supplementary Fig. 37 and Supplementary Methods).

We compared our genotyped set to other SV sets for the 1kGP cohort, including the HPRC PanGenie genotypes that we produced previously^1^, as well as the 1kGP short-read high-coverage SV callset (1kGP-HC)^29^ (Supplementary Figs. 38 and 39). On average, we found 26,115 SVs per genome, whereas this number was 18,462 for the HPRC genotypes and 9,596 for the 1kGP-HC SV calls. We specifically observed increases for rare variants (allele frequency < 1%; Fig. 2d). While the average number of rare SVs per genome was 87 for non-African individuals in the HPRC set and 169 in the 1kGP-HC set, we can now access on average 362 rare alleles. For African individuals, we detected 1,490 rare SVs per genome, whereas there were 382 previously for the HPRC and 477 for the 1kGP-HC set.

### Personal genome reconstruction

Next, we asked to what extent our improved genotyping abilities allow us to reconstruct the full haplotypic sequences of genomes sequenced with short reads. To this end, we combined our filtered PanGenie genotypes with rare SNP and indel calls obtained from Illumina reads for all 3,202 1kGP individuals (Methods) and phased this combined set using SHAPEIT5 (Supplementary Fig. 31, step 3, and Supplementary Figs. 40 and 41; see Methods).

**Extended Data Fig. 4:**
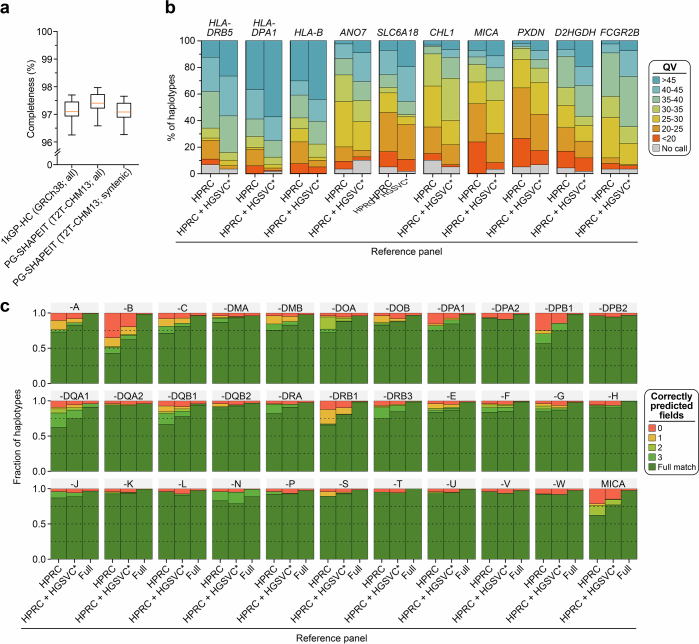
Genotyping from short-read sequencing data. **a**) Completeness statistics for haplotypes produced from the 1kGP-HC phased set (GRCh38-based) and by genome inference with Pangenie followed by phasing (T2T-CHM13–based). To allow for comparison between the GRCh38- and T2T-CHM13-based callsets, we additionally restricted our analysis to “syntenic” regions of T2T-CHM13, i.e., excluding regions unique to T2T-CHM13. For both phased sets, completeness was computed on a subset of n = 30 individuals. The median is marked in yellow, and the lower and upper limits of each box represent lower and upper quartiles (Q1 and Q3). Lower and upper whiskers are defined as Q1 − 1.5(Q3–Q1) and Q3 + 1.5(Q3–Q1). **b**) Locityper genotyping accuracy for 10 target loci with the highest average variant-based QV improvement. **c**) Locityper genotyping results for HLA genes on 61 Illumina short-read HGSVC datasets using three reference panels: HPRC (90 haplotypes), leave-one-out HPRC + HGSVC (HPRC + HGSVC*, 214 haplotypes), and HPRC + HGSVC (full, 216 haplotypes). Accuracy is evaluated as the number of correctly identified allele fields in the corresponding gene nomenclature.

We produced consensus haplotype sequences for all 3,202 individuals (6,404 haplotypes) by implanting the phased variants into T2T-CHM13 (only chromosomes 1–22 and X chromosome) and compared with consensus haplotypes produced from the GRCh38-based phased 1kGP-HC panel^29^. While the median *k*-mer-based quality value of the long-read assemblies was 53, we observed a median *k*-mer-based quality value of 45 for the consensus haplotypes computed from our short-read-based phased genotypes (Fig. 2e and Supplementary Fig. 42). To enable a fair comparison with the GRCh38-based 1kGP-HC consensus haplotypes, we additionally computed our *k*-mer-based quality value estimates restricted to regions shared between T2T-CHM13 and GRCh38 (‘CHM13-syntenic’). For these regions, we observed a median quality value of 48, whereas the quality value for the 1kGP-HC set was lower (median of 43; Fig. 2e and Supplementary Fig. 42). In addition, we observed higher *k*-mer completeness values (median of 97.4%) than for the 1kGP-HC-phased set (median of 97.1%; Extended Data Fig. 4a and Supplementary Fig. 42). Because *k*-mer-based quality value estimates do not fully capture structural sequence correctness, we additionally used PAV to compute variant-calling-based quality value estimates for each 1-Mb genomic window (Methods). This expectedly resulted in lower quality value estimates (median quality value for 1kGP-HC of 26.7; median quality value for PanGenie of 34.2), but confirms the gain of PanGenie over standard short-read pipelines (Supplementary Figs. 43–45). Of note, PanGenie enables an accurate genome reconstruction of quality value > 30 routinely (78% of all 1-Mb windows), whereas that is rarely achieved for the 1kGP-HC callset (24% of all 1-Mb windows).

### Targeted genotyping of complex loci

Although PanGenie performed well in this genome-wide setting, its use of *k*-mer information could make it difficult to genotype complex, repeat-rich loci with few unique *k*-mers. We therefore used the targeted method Locityper^30^ to genotype the 1kGP cohort across 347 polymorphic targets covering 18.2 Mb and 494 protein-coding genes (Methods), including 268 challenging medically relevant genes^31^. For this challenging set of regions, the 1kGP-HC callset reaches a variant-based quality value of 30 for only 34.5% and a variant-based quality value of 40 for only 12.8% of predictions^30^.

The performance of Locityper is constrained by the haplotypes available in the reference set. Therefore, we first evaluated haplotype availability by comparing sequences of the unrelated assembled haplotypes. Across all target loci, 51.5% of our assembled haplotypes were similar (variant-based quality value ≥ 40) to some other haplotype from the full reference panel described above, compared with only 39.6% of haplotypes when restricting to an HPRC-only reference panel^1^ (Fig. 2f).

The increased haplotype availability translates into improved genotyping of polymorphic loci and we observed 80.0% haplotypes to be predicted with variant-based quality value ≥ 30 using a leave-one-out experiment compared with 74.6% haplotypes for the HPRC-only panel (Methods). These global improvements are mirrored by improvements of individual genes (Extended Data Fig. 4b), including *HLA-DRB5*, *HLA-DPA1* and *HLA-B* (Extended Data Fig. 4c). Finally, we asked what performance could potentially be achieved for growing reference panels and therefore used the full reference panel, including samples to be genotyped. Here Locityper predicts haplotypes with average quality value of 45.8, suggesting that sequence resolution of more reference haplotypes will aid future re-genotyping of challenging medically relevant genes, with applications to disease cohorts.

## Major histocompatibility complex

**Fig. 3:**
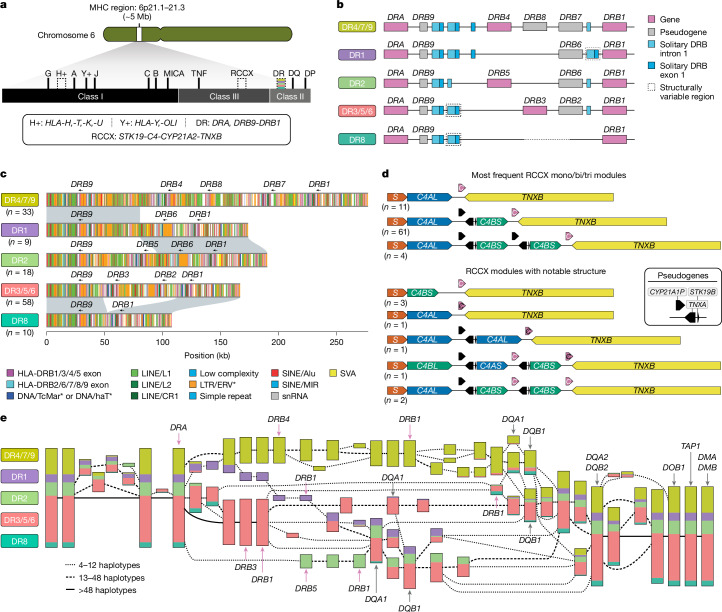
Structurally variable regions of the MHC locus. **a**, Overview of the organization of the MHC locus into class I, class II and class III regions and the genes contained therein. Structurally variable regions are indicated by dashed lines. The coloured stripes show the approximate location of the regions analysed in **b**–**d**. **b**, Gene content and locations of solitary *HLA-DRB* exon 1 and intron 1 sequences in the HLA-DR region of the MHC locus by the DR group, an established system for classifying haplotypes in the HLA-DR region according to their gene or pseudogene structure and their *HLA-DRB1* allele. **c**, High-resolution repeat maps and locations of gene or pseudogene exons for different DR group haplotypes in the HLA-DR region, highlighting sequence homology between the DR1 and DR4/7/9 and DR2, and between the DR8 and DR3/5/6, haplotype groups, respectively. Also shown is the number of analysed MHC haplotypes per DR group. CR1, chicken repeat 1; ERV, endogenous retrovirus; MIR, mammalian interspersed repeat; snRNA, small nuclear RNA. **d**, Visualization of common and notable RCCX haplotype structures observed in the HGSVC MHC haplotypes, showing variation in gene and pseudogene content as well as the modular structure of RCCX (*STK19* (S), non-functional *CYP21A2* (black C), functional *CYP21A2* (white C) and *C4L*/*S* (long ((HERV-K insertion)/short(no HERV-K insertion))). **e**, Visualization of a PGR-TK analysis of 55 MHC loci and T2T-CHM13 for 111 haplotypes in total. The colours indicate the relative proportion of distinct DR group haplotypes flowing through the visualized elements.

Given the disease relevance and complexity of the 5-Mb MHC region^32–34^ (Fig. 3a), we annotated 27–33 human leukocyte antigen (HLA) genes and 140–146 non-HLA genes or pseudogenes along with the associated repeat content of the 130 complete or near-complete MHC haplotypes (Supplementary Table 47). While 99.2% (357 of 360) of the HLA alleles agree with classical typing results^35^ (Supplementary Tables 48 and 49), we resolved a total of 826 incomplete HLA allele annotations in the IPD-IMGT/HLA reference database^36^ (Supplementary Table 50), including 112 sequences from the HLA-DRB loci, important for vaccine response and autoimmune disease^37,38^. We detected 170 SVs absent from reported reference haplotypes^39,40^ (Supplementary Table 51), including a deletion of *HLA-DPA2* (HG03807, haplotype 1).

The observed MHC class II haplotypes reflect the established DR group system (Fig. 3b and Supplementary Table 52) and comprise representatives of DR5, DR8 and DR9, which have not previously been analysed in detail^39,40^. In this system, the functional DRB3, DRB4 and DRB5 genes differentially associate across the DR groups, with DR1 and DR8 groups uniquely lacking either of them. Repeat element analyses (Supplementary Figs. 46–48; see Methods) suggest that DR8 arose from an intrachromosomal deletion mediated by 150 bp of sequence homology between *HLA-DRB1* and *HLA-DRB3* on the DR3/5/6 haplotype, as previously reported^41^ (Fig. 3c). DR1 is most likely derived by recombination between DR2 and DR4/7/9 (Fig. 3c and Supplementary Figs. 46 and 49). Finally, our catalogue of solitary *HLA-DRB* exon sequences^42^ includes refined copy number estimates (for example, three solitary *HLA-DRB* exon 1 sequences instead of one in the *HLA-DRB9* region of DR1), as well as identification of a polymorphic, solitary exon 10 kb 3′ of *HLA-DRB1* (Fig. 3b; see Methods).

Similarly, we characterized the RCCX (*STK19* (R), *C4* (C), *CYP21* (C) and *TNX* (X)) multi-allelic cluster (Fig. 3d, Supplementary Table 53 and Supplementary Fig. 50), in which phasing and variant classification has been challenging due to extensive sequence homology^43^. Tandem duplications (aka RCCX bi-modules) are the most abundant (74.6% or *n* = 97), with mono-modules and tri-modules comparable in frequency (13.1% (*n* = 17) and 12.3% (*n* = 16), respectively; Supplementary Fig. 50). Resolved haplotypes also facilitate the detection of interlocus gene conversion events critical for RCCX evolution^44^, such as two haplotypes with a tri-modular RCCX with two functional *CYP21A2* copies, one mono-modular and one bi-modular haplotype with no functional *CYP21A2* genes; and one tri-modular haplotype with a unique configuration where *C4B* precedes *C4A* and carries two *CYP21A2* copies, one of which being non-functional (Fig. 3d). We suggest that the latter haplotype was generated by introduction of a nonsense mutation and two gene conversion events, converting *CYP21A1P* into *CYP21A2* and *C4A* into a *C4B* that now unusually encodes the Rodgers blood group epitope. We also identified seven novel *C4* amino acid variants (Supplementary Figs. 51 and 52).

Next, we evaluated the performance of Locityper across 19 MHC protein-coding genes and 14 pseudogenes. Across all 33 loci, Locityper correctly predicted gene alleles in 81.0% cases when restricting to a limited HPRC-only reference panel (45 individuals)^1^. Inclusion of our assemblies (*n* = 107 individuals or 214 phased haplotypes) increased accuracy to 86.3% (leave-one-out experiment) and 97.1% (full panel leveraging all 214 phased haplotypes; Extended Data Fig. 4c), underscoring the value of accurate phased assemblies for the interpretation of short-read data.

Finally, we tested whether the established HLA class II DR group nomenclature could be recapitulated using unbiased, sequence-based analysis. Applying a pangenomic multiscale approach, PGR-TK^45^ (Fig. 3e), to a subset of our genomes (*n* = 55) as well as T2T-CHM13 (ref. ^4^), we identified 63 conserved blocks greater than 6 kb. Multiscale hierarchical clustering of the haplotypes perfectly reconstituted the traditional DR group system in the region around *HLA-DRB1* (Fig. 3e). However, we also observed additional diversified subgroups indicating the possibility for a more fine-grained future classification of HLA-DR haplotypes or utility in the context of GWAS, especially when coupled with the improved targeted genotyping ability (Extended Data Fig. 4c).

## Complex structural polymorphisms

**Fig. 4:**
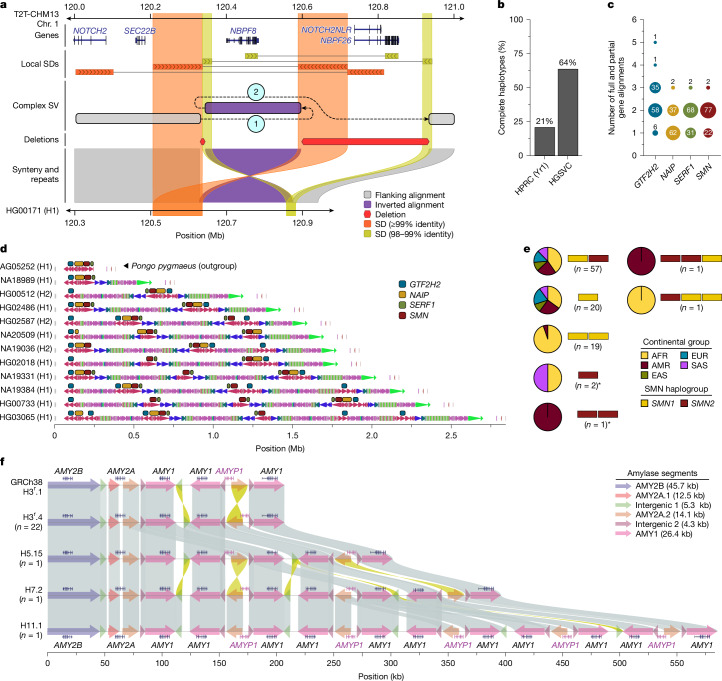
Complex SVs in human populations. **a**, An SD-mediated CSV inverts *NBPF8* and deletes *NOTCH2NLR* and *NBPF26*. Inverted SD pairs (orange and yellow bands) each mediate a template switch (dashed lines ‘1’ and ‘2’). PAV refines alignment artefacts in large repeats surrounding CSVs to obtain a more accurate representation of these structures. The allele shown is HG00171 haplotype 1. **b**, Fraction of all assemblies having complete and accurate sequence over the SMN region, stratified by study (HPRC-Yr1 and HGSVC). **c**, Copy number (full and partial gene alignments) of each multi-copy gene (*SMN1/2* in red, *SERF1A/B* in green, *NAIP* in gold and *GTF2H2/C* in blue) across assembled haplotypes (*n* = 101). **d**, SMN duplications from 11 diverse human haplotypes assembled from this study, the HPRC (HG02486) and one *Pongo pygmaeus* haplotype (top) used as an outgroup. **e**, Summary of *SMN1* (yellow) and *SMN2* (red) gene copies genotyped across human haplotypes (*n* = 101). The yellow and red bars show a unique copy number of *SMN1* and *SMN2*, whereas the pie charts show their relative proportions in continental groups. The asterisks show haplotypes with only *SMN2* gene copies. **f**, The structure of the human amylase locus shows amylase genes (coloured arrows) and alignments between haplotypes (99–100% sequence identity). The H3^r^.4 haplotype represents the most common haplotype, H5.15 and H7.2 are haplotypes previously unresolved at the base-pair level, and H11.1 is a previously unknown haplotype. Amylase gene annotations are displayed above each haplotype structure. The structure of each amylase haplotype, composed of amylase segments, is indicated by the coloured arrows. Sequence similarity between haplotypes ranges from 99% to 100%.

Long-read-assembled genomes significantly enhance the detection and characterization of complex structural variants (CSVs) defined here as a single event composed of simple SVs spanning more than one repair junction. Because CSV breakpoints are often located in repetitive sequences, including SDs and MEIs^46–49^, we recently updated PAV^8^ to identify CSVs embedded in large complex repeats such as SDs (Methods). Using this method against the T2T-CHM13 reference genome, we found on average 72 CSVs per genome^50^ (range of 51–91; Supplementary Table 54; see Data availability). Across all genomes, we identified 1,247 CSVs with 128 distinct complex reference signatures^50^, consistent with known CSVs derived from diverse individuals^51^. We found that 27% of CSVs have locally duplicated sequences, and 38% have local inversions. Many of the complex structures that we identified are mediated by SDs, such as INVDUP-INV-DEL (174 CSVs and 92% SDs), DEL-INV-DEL (34 CSVs and 21% SDs) and INVDUP-INV-INVDUP (8 CSVs and 75% SDs) where DEL is a reference deletion, INV is an inverted sequence that is not duplicated and INVDUP is a duplicated inversion (one copy in each orientation)^50^. As an example, we highlight two CSVs involving *NOTCH2NL* and *NBPF*, genes implicated in the expansion of the human brain during evolution^8^, as well as a core duplicon associated with genomic instability^52^. Although the full structures could not be resolved by previous optical mapping or sequencing experiments, we can distinguish three distinct haplotype structures, including a reference haplotype (13.7% allele frequency), a 930-kb CSV (DEL-INV-DEL) inverting *NBPF8* and deleting *NOTCH2NLR* and *NBPF26* (35.9% allele frequency; Fig. 4a), and a 513-kb CSV with a distal template switch replacing *NBPF8* with *NBPF9* (50.8% allele frequency; Supplementary Fig. 53).

As a second example, the structurally complex region containing *SMN1* and *SMN2* gene copies is associated with spinal muscular atrophy, and successful ASO-mediated gene therapies involve *SMN2* (refs. ^53,54^). The genes are embedded in a large SD region (approximately 1.5 Mb) that has been almost impossible to fully sequence resolve despite the advances of the past two decades^1,2,8^ (Supplementary Fig. 54). We successfully assembled, validated and characterized 101 haplotypes to fully resolve the structure and copy number of *SMN1/2*, *SERF1A/B*, *NAIP* and *GTF2H2/C* (Methods). We found that 48% (*n* = 48) of haplotypes carry exactly two copies of *SMN1/2*, *SERF1A/B* and *GTF2H2/C*, whereas *NAIP* is present mostly in a single copy. We highlight 11 human haplotypes showing increasing complexity (Fig. 4b–d). We specifically distinguished functional *SMN1* and *SMN2* copies based on our assemblies (Supplementary Fig. 55) and compared them with the short-read-based genotyping methods Parascopy and SMNCopyNumberCaller (Methods). For individuals with two fully assembled haplotypes (*n* = 31), predicted *SMN1/2* copy numbers matched perfectly among the three methods (Supplementary Fig. 56). Our analysis shows that 98 haplotypes carry the ancestral *SMN1* copy but three do not and are potentially disease-risk loci that may have arisen as a result of interlocus gene conversion (Fig. 4e and Supplementary Fig. 57).

Finally, we analysed the complex amylase locus spanning 212.5 kb on chromosome 1 (GRCh38; chr. 1: 103554220–103766732) and containing genes *AMY2B*, *AMY2A*, *AMY1A*, *AMY1B* and *AMY1C*^55^ (Fig. 4f). From 65 sequence-resolved genomes, we identified 39 distinct amylase haplotypes, capturing approximately 83% of the haplotypes in the population (Supplementary Table 55 and Supplementary Figs. 58 and 59), 35 of which were supported by both Verkko and optical genome mapping de novo assemblies. The length of these amylase haplotypes ranges from 111 kb (H1^a^.1 and H1^a^.2) to 582 kb (H11.1; Fig. 4f), including those that are structurally identical to the GRCh38 (H3^r^.1) and T2T-CHM13 (H7.3) assemblies. Among these, four are common: H1^a^.1 (*n* = 14), H3^r^.1 (*n* = 13), H3^r^.2 (*n* = 19) and H3^r^.4 (*n* = 22; constituting 57% of all genomes), whereas 23 are singletons. We identified nine haplotypes previously supported only by optical genome mapping data and fully sequence resolved the largest haplotype (H11.1; 11 *AMY1* (8.8 kb) copies)^55–57^ (Fig. 4f).

## Centromeres

**Extended Data Fig. 5:**
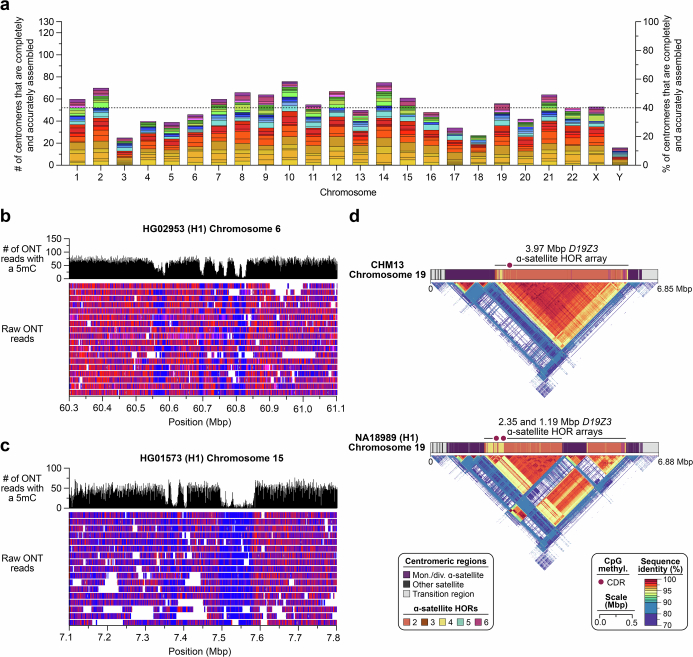
Assembly of 1,246 human centromeres across 65 diverse human genomes show genetic and epigenetic variation. **a**) Number (left y-axis) and percentage (right y-axis) of centromeres that are completely and accurately assembled among 65 diverse human genomes, colored by population group. Mean, dashed line. **b,c**) Examples of di-kinetochores, defined as two CDRs located >80 kbp apart from each other, on the **b**) HG02953 chromosome 6 centromere and **c**) HG01573 chromosome 15 centromere. UL ONT reads span both CDRs in each case, indicating that the CDRs occur on the same chromosome in the cell population. **d**) Differences in the α-satellite HOR array organization and methylation patterns between the CHM13 and NA18989 (H1) chromosome 19 centromeres. The NA18989 (H1) chromosome 19 centromere has two CDRs, indicating the potential presence of a di-kinetochore.

Human centromeres are among the most mutable genomic regions and are composed of tandemly repeating α-satellite DNA organized into higher-order repeats (HORs) spanning up to several megabases on each chromosome^58^. It has been estimated that approximately 22% of centromeres vary by over 1.5-fold in length, and approximately 30% of them vary in their structure^59^. To understand the genetic and epigenetic centromeric variation in these 65 individuals, we first assessed contiguity and accuracy using two assembly algorithms (Methods). We identified 822 Verkko centromeres and 777 hifiasm centromeres that were completely and accurately assembled. Only 28.3% were correctly assembled by both assemblers, with Verkko and hifiasm uniquely resolving a similar subset (37.7% and 34.1%, respectively). We combined these two datasets into a non-redundant set of 1,246 completely and accurately assembled centromeres (approximately 52 centromeres per chromosome and approximately 19.5 centromeres per genome, on average; Extended Data Fig. 5a and Supplementary Tables 56 and 57).

**Fig. 5:**
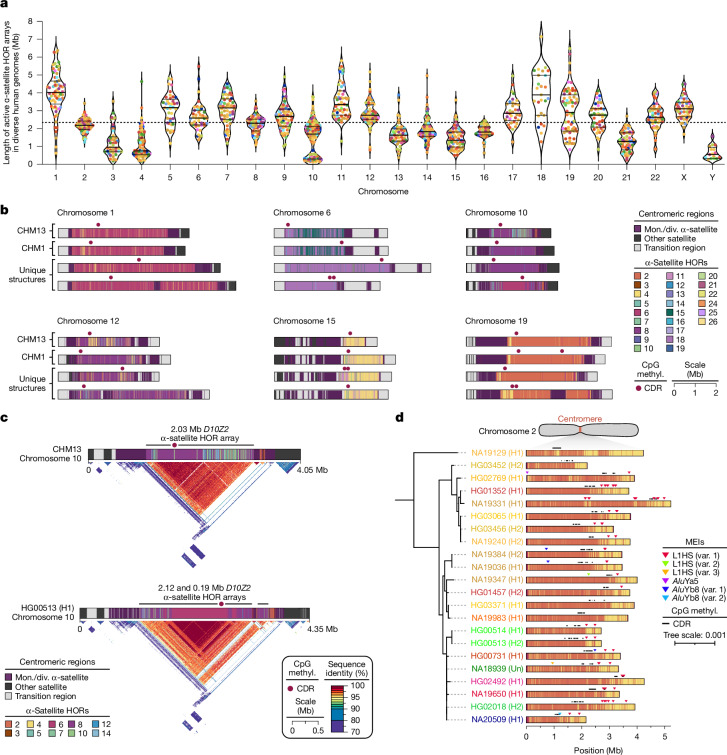
Variation in the sequence, structure and methylation pattern among 1,246 human centromeres. **a**, Length of the active α-satellite HOR array (arrays) for each complete and accurately assembled centromere from each genome. Each data point indicates an active α-satellite HOR array and is coloured by population group. The median length of all α-satellite HOR arrays is shown as a dashed line. For each chromosome, the median (solid line) and first and third quartiles (dashed lines) are shown. **b**, Sequence, structure and methylation (methyl.) map of centromeres from CHM13, CHM1 and a subset of 65 diverse human genomes. The α-satellite HORs are coloured by the number of α-satellite monomers within them, and the site of the putative kinetochore, indicated by the CDR, is shown. Mon., monomeric; div., divergent. **c**, Differences in the α-satellite HOR array organization and methylation patterns between the CHM13 and HG00513 (H1) chromosome 10 centromeres. The CDRs are located on highly identical sequences in both centromeres, despite their differing locations. **d**, MEIs in the chromosome 2 centromeric α-satellite HOR array. Most MEIs are consistent with duplications of the same element rather than distinct insertions, and all of them reside outside of the CDR. Var., variant.

We first measured the variation in the length of the centromeric α-satellite HOR array (or arrays) on each chromosome. Although active centromeric α-satellite HOR arrays are, on average, 2.3 Mb in length, there is considerable variation, including outliers (Fig. 5a, Supplementary Table 57 and Supplementary Figs. 60 and 61). For example, the active α-satellite HOR arrays from chromosomes 3, 4, 10, 13–16, 21 and the Y chromosome are consistently smaller, whereas those on chromosomes 1, 11 and 18 are larger than average (Supplementary Fig. 61). Among the 1,246 centromeres, we identified 4,153 new α-satellite HOR variants and novel active α-satellite HOR array organizations (Fig. 5b and Supplementary Figs. 62 and 63). On chromosome 1, for example, we identified an insertion of monomeric α-satellite into the *D1Z7* α-satellite HOR array, effectively splitting the α-satellite into two distinct HOR arrays (Fig. 5b). A similar bifurcation event also occurred on the centromeres of chromosomes 12 and 19, generating two α-satellite HOR arrays where there typically is only one (Fig. 5b,c). In addition, we found novel α-satellite HOR array organizations for chromosomes 6 and 10 that differ from the CHM1 and CHM13 arrays on those chromosomes^59^ (Fig. 5b and Supplementary Fig. 62b,c). These array organizations, which are the most common in our dataset, are primarily composed of either 18-monomer α-satellite HORs (chromosome 6) or 6-monomer α-satellite HORs (chromosome 10).

To determine how variation in centromeric sequence and structure affects their epigenetic landscape, we assessed the CpG methylation pattern along each centromere using native ONT data. We found that all centromeres contain at least one region of hypomethylation (termed the ‘centromere dip region’ (CDR))^58,60^, which is thought to mark the site of the kinetochore. However, in many cases, such as on chromosomes 6, 15 and 19, there were at least two CDRs more than 80 kb apart (Fig. 5b, Extended Data Fig. 5b–d and Supplementary Fig. 64). This suggests the presence of a ‘di-kinetochore’, which may form a dicentric chromosome on approximately 7% of chromosomes, but additional analyses that assess the location of the centromeric histone H3 variant, CENP-A, will need to be performed to confirm these putative kinetochore sites. We generated sequence identity heatmaps of each centromere and found that the CDR often resides within the most highly identical regions of the α-satellite HOR arrays (Fig. 5c and Extended Data Fig. 5d). Even when the α-satellite HOR array is split into two arrays, such as on chromosome 19, the CDR associates with the array containing some of the most highly identical α-satellite HORs (Extended Data Fig. 5d). This suggests that the kinetochore may track with actively homogenizing α-satellite HOR sequences in response to a co-evolution between centromeric DNA and proteins^61^.

MEI investigation in many of the α-satellite HOR arrays (Methods) revealed that approximately 30% contained at least one MEI. In total, we identified 89 unique polymorphic insertions with varying allele frequencies (Supplementary Table 58), with L1HS being the most prevalent (58%), followed by *Alu* elements (41%) and SVAs (1%). The *D2Z1* α-satellite HOR array on chromosome 2 was highly enriched with MEIs (Fig. 5d), with at least one L1HS and/or *Alu* insertion in 80% of haplotypes (Supplementary Fig. 65). Although L1HS insertions or duplications were the most common, occurring on average three times per array, three unique *Alu* insertions (two *Alu*Yb8 and one *Alu*Ya5) were also present, albeit with low allele frequency. Nearly all insertions, as well as their duplications, were located outside of the CDRs and typically towards the periphery. However, one *Alu*Yb8 insertion (NA20509 (H1)) was located between two CDRs and appeared to ‘break’ a single CDR into two, whereas a pair of L1HSs were found on either side of a CDR in two haplotypes (NA19331 (H1) and NA19650 (H1)), possibly acting as boundaries that restrict CDR and CENP-A chromatin movement, as previously suggested^62^.

## Discussion

LRS and assembly have enabled both the full resolution of a human genome sequence^4^ and fundamentally deepened our understanding of human genetic diversity^1,8,13,63^. The development of a human pangenome reference^1,64^ requires ideally completely phased and assembled diverse genomes. Although hundreds of genomes are being assembled as part of international efforts^65^, practically, few are yet truly T2T. Meanwhile, pangenome augmentation methods based on shallow long-read data have been used to capture variants with lower allele frequencies^66^. Nevertheless, algorithms and technology have advanced significantly, and we have demonstrated that more than 99% of the human genome can be accurately phased and assembled by focusing on 65 diverse humans (130 haplotypes). We characterized regions previously excluded or collapsed^1,2^, including centromeres, biomedically complex regions such as *SMN1/SMN2*, the MHC and thousands of more complex SV patterns.

Combining our assemblies with previous HPRC assemblies to create a reference set, we were able to reconstruct a genome from short reads to an average base error of about 0.00158% (quality value of 48). This process detects 26,115 SVs per genome on average from short-read sequence data and notably now recovers more rare SVs (allele frequency < 1%) than direct variant discovery from short reads. This advance was made possible by improvements in assembly quality, the larger sample size, improved versions of the Minigraph-Cactus and PanGenie applications, and the switch to the more complete T2T-CHM13 reference genome. As the number of HPRC genomes increases to several hundreds and they reach T2T status^65^, genotyping accuracy will probably improve further. This, in turn, will make disease-association studies from short reads considerably more powerful for complex variation.

Using our assembly method, we fully assembled 1,246 centromeres — 42% of all possible centromeres in these individuals. As expected, we observed considerable variation in the content and length of the α-satellite HOR array (up to 37-fold for chromosome 10) consistent with its higher mutation rate and more rapid evolutionary turnover^2,59^. We have also documented recent *Alu*, L1 and SVA retrotransposition into the α-satellite HORs and showed that these may be used to tag HOR expansions on particular human haplotypes. Using the CDR^58,60^ as a marker of kinetochore attachment, we have shown considerable variation in the location across human centromeres and remarkably that 7% of human chromosomes show evidence of two or more putative kinetochores (that is, di-kinetochores) in lymphoblastoid cell lines. The significance of both MEIs and di-kinetochore on chromosome segregation or missegregation will need to be experimentally assessed, and these phased genomes (and their corresponding cell lines) provide the foundation for such future work.

Finally, from a technical perspective, application of two independent assembly algorithms, hifiasm (ultra-long) and Verkko, nearly doubled the number of sequence-resolved centromeres. Although the two methods were strongly complementary for centromeres, Verkko was clearly superior for the Y chromosome (Supplementary Fig. 26c). As the performance of both Verkko and hifiasm has been shown to be very similar for large portions of the euchromatin^10^, there is benefit in applying both assembly algorithms to resolve the most structurally complex regions of the genome until a tool combining the strengths of both methods becomes available.

## Methods

### Sample selection

A total of 65 diverse humans were included in the current study. The majority of the individuals (63 of 65) originated from the 1kGP sample set^11^, one (NA21487) from the International HapMap Project^67^ and one (NA24385, also called HG002) commonly used for benchmarking by the Genome in a Bottle (GIAB) Consortium^68^ was included in all analyses with publicly available data from other efforts (Supplementary Tables 1–4, 6 and 7). Individuals were selected to maximize genetic diversity and Y chromosome lineages (Supplementary Methods).

### Data production

In addition to data generated through previous efforts^8,23^, sequencing libraries were prepared from high-molecular-weight DNA or RNA extracted from lymphoblastoid cell lines (Coriell Institute). PacBio HiFi sequencing data were generated on the Sequel II or Revio platforms using 30-h movie times. UL ONT libraries were generated using a modified fragmentase protocol and sequenced on R9.4.1 flow cells on a PromethION instrument for 96 h. Bionano Genomics optical mapping data using DLE-1 tagging were collected on Saphyr 2nd generation instruments. Strand-seq data were produced using BrdU incorporation and second-strand DNA removal during PCR-based library construction to generate single-nucleus barcoded libraries sequenced on an Illumina NextSeq 500 platform^69,70^. Hi-C data were collected using Proximo Hi-C kits (v.4.0; Phase Genomics) and sequenced on an Illumina NovaSeq 6000. RNA-seq libraries were generated using KAPA RNA Hyperprep with RiboErase (Roche) and sequenced on an Illumina NovaSeq 6000 platform. Iso-Seq full-length cDNA libraries were created with the Iso-Seq Express protocol and sequenced on a PacBio Sequel II system. Detailed descriptions of materials and methods are available (Supplementary Methods).

### Assembly

We produced fully phased hybrid assemblies using Verkko (v.1.4.1)^9^ as our primary assembler (Supplementary Methods). We additionally created hifiasm (ultra-long; v.0.19.6)^10^ assemblies (Supplementary Methods), which were used to complement our analysis of the most challenging regions (centromeres and Yq12). The phasing signal for all assemblies was generated using the Graphasing pipeline^12^ (v.0.3.1-alpha). All assemblies were scanned for contamination with NCBI’s Foreign Contamination Screening workflow (v.0.4.0)^71^ and annotated for potential assembly errors using Flagger (v.0.3.3)^1^, Merqury (v.1.0)^72^, NucFreq^73^ (commit #bd080aa) and Inspector (v.1.2)^74^ (Supplementary Methods). Assembly quality was assessed by computing quality value estimates with Merqury and DeepVariant (v.1.6)^75^ as previously described^8^. Gene completeness of the assemblies was evaluated using compleasm (v.0.2.5)^76^ and the primate set of known single-copy genes of OrthoDB (v.10)^77^. The T2T status of the assembled chromosomes and the closing status of previously reported gaps^2^ were determined relative to the T2T-CHM13 reference genome^4^ by factoring in the above quality control information in the evaluation of the contig-to-reference alignment produced with minimap2 (v.2.26)^78,79^ and mashmap (v.3.1.3)^80^ (Supplementary Methods). The parental support for the assembled child haplotypes in the three family trios was computed by evaluating the CIGAR operations in the minimap2 contig-to-contig alignments between the parents and child.

### Variant calling

#### Genome reference

Callsets were constructed against two references: GRCh38 (GRCh38-NoALT) and T2T-CHM13 (T2T-CHM13v.2.0)^4^.

#### Variant discovery and merging

For assembly-based callsets, we ran PAV (v.2.4.1)^8^ with minimap2 (v.2.26)^78^ and LRA (v.1.3.7.2)^81^ alignments, DipCall (v.0.3)^82^ and SVIM-asm (v.1.0.3)^83^. SVIM-asm used PAV alignments before PAV applied any alignment trimming, and DipCall produced minimap2 alignments for DipCall variants (Supplementary Methods).

For PacBio HiFi callsets, we ran PBSV (https://github.com/PacificBiosciences/pbsv; v.2.9.0), Sniffles (v.2.0.7)^84^, Delly (v.1.1.6)^85^, cuteSV (v.2.0.3)^86^, DeBreak (v.1.0.2)^87^, SVIM (v.2.0.0)^88^, DeepVariant (v.1.5.0)^75^ and Clair3 (v.1.0.4)^89^. The same callers and versions were run for ONT except for PBSV, and DeepVariant was executed through PEPPER-Margin-DeepVariant (r0.8)^90^. The callset process was the same for both references (Supplementary Methods).

SV-Pop^8^ was used to merge PAV calls from minimap2 alignments and generate per-sample support information from all other callers. Calls in T2T-CHM13 were filtered if they intersected the UCSC ‘CenSat’ track for T2T-CHM13 (UCSC hs1) with monomeric (‘mon’) records excluded or if they were in telomere repeats. GRCh38 variants intersecting modelled centromeres were removed (Supplementary Methods).

### MEIs

MEIs were identified within the 130 haplotype assemblies using two separate pipelines and human references (T2T-CHM13 and GRCh38). One detection pipeline, L1ME-AID (v.1.0.0-beta; L1 Mediated Annotation and Insertion Detector; see Code availability), leverages a local RepeatMasker (v.4.1.6)^91^ installation with the Dfam (v.3.8) database^92^ to annotate the freeze4 PAV-merged SV insertion callsets (T2T-CHM13 and GRCh38). The second pipeline called MEIs directly from the alignment of contigs to a reference genome with PALMER2 (Code availability). Putative MEIs from both callers were then merged using MEI coordinates, element family (*Alu*, L1, SVA, HERV-K or snRNA) and sequence composition (Supplementary Methods). Next, MEIs were curated to distinguish MEIs from deletions (T2T-CHM13 or GRCh38), duplications or potential artefacts (for example, possible genome assembly errors; Supplementary Methods). All MEIs called by a single pipeline that passed quality control were manually curated. Finally, both callsets were compared against an orthogonal MEI callset produced by MELT-LRA (Supplementary Methods; see Code availability). To determine intact ORFs across LINE-1 elements, we followed a previously described method^8^ to detect intact ORF1p and ORF2p from full-length (more than 5,900 bp) LINE-1 insertions.

Separately, MEIs within centromere HOR arrays were identified with RepeatMasker (v.4.1.6)^91^ and the Dfam library (v.3.8)^92^, annotation of complete and accurately assembled centromeres (see ‘Centromeres’ in the Methods). The sequences of *Alu* elements, L1s and SVAs identified by RepeatMasker within the centromere HOR array boundaries were retrieved using SAMtools (v.1.15.1)^93^. Element sequences were then scrutinized with L1ME-AID (v.1.0.0-beta) utilizing the same cut-offs applied to the freeze4 PAV-merged SV insertion callset to distinguish young MEIs from older mobile element fragments. Sequence of all putative MEIs that passed filtering were re-retrieved along with a flanking sequence (±100 bp) using SAMtools (v.1.15.1)^94^, and then aligned against one another using MUSCLE (v.3.38.31)^95^ to distinguish unique MEIs from duplicated insertions of MEIs residing in centromere regions (Supplementary Table 58).

### Inversions

We performed validation of the T2T-CHM13-based and GRCh38-based PAV inversion callsets, individually, using Strand-seq-based re-genotyping of the inversion calls. Before genotyping, we performed Strand-seq cell selection using ASHLEYS^96^. The good-quality Strand-seq cells were used as input to perform genotyping by ArbiGent^16^ (Supplementary Methods).

We evaluated the PAV inversion callset for one candidate carrier per region using manual dotplot analysis with NAHRwhals^97^. NAHRwhals was applied to detect the false discovery rate and classify all candidate inversion regions larger than 5 kb into distinct inversion classes.

We compared the PAV inversion callset reported with respect to T2T-CHM13 to a previously published callset^98^ based on a subset of genomes reported in this study. Using the 25% reciprocal overlap criterion, we defined inversions detected in both callsets as well as inversions that are new to the current study. We evaluated all novel inversion candidates manually using dotplot analysis of each putative novel inversion.

### SD and copy number polymorphic genes

#### Identification of SDs

SD annotation was performed using SEDEF (v.1.1)^99^ after masking repeats (TRF (v.4.1.0)^100^, RepeatMasker (v.4.1.5)^101^ and Windowmasker (v.2.2.22)^102^; Supplementary Methods). SDs with a sequence identity of more than 90%, length of more than 1 kb, satellite content of less than 70% and free of putative erroneous regions (see Code availability) were retained. In addition, the highly confident SD callset was further validated by fastCN^103^. Comparative analysis of SDs was conducted in T2T-CHM13 space. Positions of the SDs in T2T-CHM13 were mapped as follows: (1) linking SDs within 10-kb distance, (2) identifying those SD chains that are located in alignment block of at least 100 kb in size, and (3) projecting the chained SDs onto putative homologous SD loci containing at least one 10-kb unique flank. In addition, syntenic SDs were further assessed for whether they share sequence content by aligning SDs with minimap2 (v.2.26)^78^; the following SDs were quantified: (1) SDs unobserved by T2T-CHM13, (2) having changed sequence content (less than 80% of the sequence conserved), and (3) expanded size (at least twofold).

#### Duplicated genes

Protein-coding transcripts from GENCODE v.44 (Liftoff to T2T-CHM13) were aligned to the genome assemblies (excluding NA19650, NA19434 and NA21487) using minimap2 (‘-cx asm20 -f 5000 -k15 -w10 -p 0.05 -N 200 -m200 -s200 -z10000 --secondary=yes --eqx’). The mapped genes were further filtered to exclude alignments due to nested repeats, keeping minimum length of 2 kb, percent identity of more than 90% and coverage of more than 80%. Multi-copy genes were determined by maximum gene counts greater than one. Variable copy number genes were defined by assessing the copy number across the population (at least one of the genome assemblies with different copy number; Supplementary Methods).

### Y chromosome variation

#### Construction and dating of Y phylogeny

The construction and dating of Y-chromosomal phylogeny combining the 30 males from the current study plus two males (HG01106 and HG01952 from the HPRC year 1 dataset for which contiguous Yq12 assemblies were used from^23^) were done as previously described^23^. Detailed descriptions of methods are available (Supplementary Methods). Please note that the male individual HG03456 appears to have a XYY karyotype as previously reported^29^.

#### Identification of sex-chromosome contigs

Contigs containing Y-chromosomal sequences from the whole-genome assemblies were identified and extracted for the 30 males as previously described^23^. Y assemblies for the two HPRC individuals, HG01106 and HG01952, were used from ref. ^23^.

#### Y chromosome annotation and analysis

The annotation of Y-chromosomal subregions was performed as previously described using both the GRCh38 and T2T-CHM13 Y reference sequences^23^. The centromeric α-satellite repeats for the purpose of Y subregion annotation were identified using RepeatMasker (v.4.1.2-p1)^91^. The Yq12 repeat annotations were generated using HMMER (v.3.3.2dev)^104^, and identification of *Alu* insertions was performed as previously described^23^. To maximize the number of contiguously assembled Yq12 subregions, hifiasm assemblies of this subregion were analysed from four individuals (NA19239, HG03065, NA19347 and HG00358) following manual inspection of repeat unit orientation and distance from each other in the assembled sequences (Supplementary Table 40).

Dotplots to compare Y-chromosomal sequences were generated using Gepard (v.2.0)^105^. Although we also assembled the T2T (NA24385/HG002) Y as a single contig (Supplementary Table 40), all analyses conducted here used the existing published T2T assembly^24^.

Visualization of eight completely assembled Y chromosomes (Supplementary Fig. 27) was based on pairwise alignments generated using minimap2 (v.2.26)^78,79^ with the following options: ‘-x asm20 -c -p 0.95 --cap-kalloc = 1 g -K4g -I8g -L --MD --eqx’. For visualization, alignments of less than 10 kb in length were filtered out. In addition, alignments were broken at SVs of more than 50 bp or more in size and then binned in 50-kb bins.

### SVs affecting genes

We annotated the potential effect of long-read SVs on genes using the coding transcripts and exons defined in GENCODE (v.45)^106^, as per Ensembl VEP (v.111)^107^. Long-read deletions or insertions are classified as coding overlapping events if at least one breakpoint falls within the coding exons of a gene. We considered genes that have a LOEUF score under 0.35 as intolerant to loss-of-function variants^27^. To specifically analyse the potential effect of MEIs on genes, the merged GRCh38 MEI callset was intersected with the findings from Ensembl^108^ (release 111) VEP^107^ (see transcriptional effect of SVs below). The MEIs were categorized by insertion location (for example, protein-coding exons, untranslated regions of protein-coding transcripts and non-coding exons), and within each category, the number of MEIs present, genes disrupted and transcripts affected were quantified. The Ensembl VEP nonsense-mediated decay (NMD) plugin (https://github.com/Ensembl/VEP_plugins/blob/release/112/NMD.pm) was utilized to predict which protein-coding transcripts with MEI-induced premature stop codons would escape NMD. Transcripts were further scrutinized by manually comparing the MEI location within the transcript sequence using the UCSC Genome Browser^109^. To ensure that the premature stop codon met one of the four requirements for NMD escape according to the exon-junction complex model^110^. Allele frequencies were then calculated (children of trios excluded) for the exon-disrupting MEIs. In the event of a ‘.’ (indicating misassembly) in the genotyping information, the haplotype was excluded from the calculation.

### Functional effect of SVs

#### Effects on exons and isoform

We used the Ensembl^108^ (release 111) Variant Effect Predictor^107^ with the NMD plugin (https://github.com/Ensembl/VEP_plugins/blob/release/112/NMD.pm) to screen the PAV freeze 4 callset for SVs that disrupt gene loci in the merged GRCh38 annotation (NCBI RefSeq GCF_000001405.40-RS_2023_03, Ensembl 111, GENCODE v.45). Protein-coding genes impacted by putative exon disruptions were evaluated for evidence of Iso-Seq expression (in more than 1 individual) across the 12 individuals. Isoforms associated with these SV-containing genes were screened for the presence of unreported splice variants using SQANTI3 (v.5.1.2)^111^. All isoforms of these candidate genes were aligned to GRCh38p14 using pbmm2 (https://github.com/PacificBiosciences/pbmm2; v.1.5.0) and visualized with IGV^112^ to identify variant-specific patterns. We compared all isoforms phased to variant haplotypes to known transcripts represented in RefSeq^113^, CHESS^114^ and GENCODE^106^ gene annotation databases to identify novel splice products and isoforms. MUSCLE (v.3.8.425)^95^ and Aliview^115^ were used to perform a multiple sequence alignment and visualize the multiple sequence alignment, respectively, between wild-type and variant haplotype assemblies to identify SV breakpoints.

#### Effects on gene expression

We next assessed SVs for enrichment near genes with altered expression in the 12 individuals with Iso-Seq data. Using gene expression quantifications from short-read RNA-seq data, we performed differential expression analysis using DESeq2 (v.1.38.3)^116^ between individuals who carried and did not carry each SV, supplemented with outlier expression analysis for singleton SVs (Supplementary Methods). We assessed SV overlap with multiple GENCODE v.45-derived genomic elements, such as protein-coding and pseudogene classes^106^, and ENCODE-derived candidate *cis*-regulatory elements^117^, using permutation tests to find enrichment or depletion of SVs for each annotation (Supplementary Methods).

#### Effects on chromatin structure and colocalization with GWAS hits

Among the 128 SV gene pairs (122 unique SVs associated with 98 genes) that exhibit significant differential gene expression changes in the 12 individuals with Iso-Seq data, we first filtered out SVs with missing genotypes in 6 or more out of 12 individuals. For each remaining SV, we extracted the 50 kb upstream and downstream of the annotated transcription start site position for each paired gene with corresponding insulation scores under 10-kb resolution (Supplementary Methods). For those insulated regions intersecting with more than one SV, we applied a local multi-test correction. A false discovery rate < 0.05 from the two-sided Wilcoxon rank-sum test was considered significant. We investigated the association between variants and human phenotypes or traits by intersecting SNVs, indels and SVs with SNPs identified in GWAS (GWAS summary statistics; gwas_catalog_v1.0.2-associations_e111_r2024-04-16.tsv)^118^. We used Plink (v.1.90b6.10)^119^ to examine the linkage disequilibrium between SNVs, indels and SVs with GWAS SNPs within 1-Mb window size.

### Genome-wide genotyping with PanGenie

We built a pangenome graph containing 214 haplotypes with Minigraph-Cactus (v.2.7.2)^120^ from the haplotype-resolved assemblies of 65 HGSVC individuals and 42 individuals from the HPRC^1^ and produced a CHM13-based VCF representation of the top-level bubbles of the graph that can be used as input for genotyping with PanGenie (Supplementary Methods). This was done by converted genotypes of male sex chromosomes to a homozygous representation, filtering out records for which at least 20% of haplotypes carry a missing allele (‘.’) and running our previously developed decomposition approach to detect and annotate variant alleles nested inside of graph bubbles (Supplementary Methods). We genotyped all 30,490,169 bubbles (representing 28,343,728 SNPs, 10,421,787 indels and 547,663 SVs) across all 3,202 1kGP individuals based on short reads^29^ using PanGenie (v.3.1.0)^3^ with additional parameter -a 108. We filtered the resulting genotypes based on a support vector regression approach^1,8^, resulting in 25,695,951 SNPs, 5,774,201 indels and 478,587 SVs that are reliably genotypable (Supplementary Methods).

### Personal genome reconstruction

#### Reference panel and personal genome construction

We used our filtered genotypes across all 3,202 individuals and added 70,174,243 additional rare SNPs and indels from an external short-read-based callset for the same 3,202 1kGP individuals (obtained from https://s3-us-west-2.amazonaws.com/human-pangenomics/index.html?prefix=T2T/CHM13/assemblies/variants/1000_Genomes_Project/chm13v2.0/all_samples_3202/; Supplementary Methods). We filtered out variants reported with a genotype quality below 10 and ran SHAPEIT5 (v.5.1.1)^121^ phase_common to phase this joint callset. We used the resulting reference panel to reconstruct personal genomes for all 3,202 individuals by implanting phased variants into the CHM13 reference genome with BCFtools^93^ to create the 6,404 consensus haplotype sequences of all 1kGP individuals (Supplementary Methods).

#### Evaluation

For the evaluation of the consensus haplotypes produced from 1kGP and PG-SHAPEIT phased genotypes, PAV was run with one of the consensus haplotypes as a reference and the other one as a query sequence, together with the respective haplotype assemblies for the same individual. We analysed the resulting variant calls to determine all variant positions with conflicting genotypes between the consensus and assembly haplotypes. For such erroneous variant positions, we then counted the number of base-pair changes in both consensus haplotypes within windows of 1 Mb in length along the reference haplotype and computed a quality value estimate as: −10 × log_10_(bp_changes/(2 × window_size)). In addition, we also counted the number of erroneous variants more than 20 bp in each window. We then plotted the distributions of these two metrics and computed the median (Supplementary Figs. 43–45b,c). We evaluated consensus sequences for a second individual (HG01114) to verify consistency of results across individuals. For each individual, we ran the experiment twice, using either haplotype as a reference sequence. In addition to evaluating the consensus haplotypes, we repeated the same experiment for HG002, using the Q100 assemblies (https://github.com/marbl/HG002) as reference sequences to align to, and our HGSVC3 assemblies as queries (Supplementary Figs. 44 and 45d). To get a baseline estimate, we also ran the experiment using CHM13 as a reference and two copies of GRCh38 as well as another copy of CHM13 as query sequences (Supplementary Fig. 45a).

### Targeted genotyping of complex polymorphic loci

Targeted genotyping was performed using Locityper (v.0.15.1)^30^ across 347 complex polymorphic target loci (Supplementary Methods). On the basis of the input short-read whole-genome sequencing data, at each of the targets, Locityper aims to identify two haplotypes from the reference panel that are most similar to the input data. Three reference panels were used: HPRC haplotypes (90 haplotypes); HPRC + HGSVC3 haplotypes (216 haplotypes); and leave-one-out HPRC + HGSVC3 panel (leave-one-out evaluation; 214 haplotypes), where two assemblies corresponding to the input dataset were removed. To evaluate prediction accuracy, we constructed sequence alignments between actual and predicted haplotypes and estimated variant-based quality values (Supplementary Methods). Locityper accuracy is limited by haplotypes present in the reference panel; consequently, we evaluated haplotype availability quality value as the highest Phred-scaled sequence divergence between actual assembled haplotypes and any haplotype from the reference panel (Supplementary Methods).

### MHC

#### Gene annotation

Immuannot-based HLA types were compared in two-field resolution to the HLA typing published earlier and obtained with PolyPheMe^35^ (http://ftp.1000genomes.ebi.ac.uk/vol1/ftp/data_collections/HLA_types/20181129_HLA_types_full_1000_Genomes_Project_panel.txt). Of the 130 haplotypes, 58 were not in the PolyPheMe dataset and were excluded. In addition to Immuannot (MHC reference version: IPD-IMGT/HLA-v.3.55.0)^36^, haplotypes were annotated using MHC-annotation v.0.1 (see Code availability). Cases of overlapping genes were resolved after inspection by removing superfluous annotations. Reported gene counts for HLA genes and C4 annotation were based on Immuannot.

#### SV detection

To search for structural variation in the DRB gene region, HGSVC MHC haplotypes were cut from (start of DRA) to (end of DRB1 + 20 kb). The coordinates were obtained using MHC-annotation v.0.1. On the basis of their DRB1 allele as determined by Immuannot (see above), the sequences were grouped into DR groups. Within each group, every sequence was aligned with nucmer^122^ (v.3.1; -nosimplify -maxmatch) to the same sequence (arbitrarily selected as the sequence with the alphanumerically smallest ID) and plotted with a custom gnuplot script based on mummerplots output. Sequences were annotated as follows: (1) repeat elements were masked with RepeatMasker (v.4.1.2)^91^; (2) full DRB genes and pseudogenes were searched for with minimap (v.2.26; ‘--secondary=no -c -x --asm10 -s100’) by aligning the sequence from against all DRB alleles from IMGT and the larger *DRB9* sequence Z80362.1 (results were highlighted and masked for the next step); (3) DRB exons were searched for with BLASTN (v.2.14.1)^123^ by aligning all DRB exons from IMGT to the sequence and filtering for highest matches (results were highlighted and masked for the next step); and (4) as for step 3 but with introns.

For each HGSVC MHC haplotype, SVs were called with PAV^8^ against eight completely resolved MHC reference haplotypes^39,40^. To determine which SVs in the HGSVC haplotypes were not present in any of the eight reference haplotypes, for each HGSVC haplotype, the ‘query’ coordinates (that is, the coordinates of the calls relative to the analysed HGSVC haplotype) of the PAV calls were padded with 50 bp on each side and the intersection of SV calls (based on the padded query coordinates, across the eight MHC reference sequences) was computed. Only variants longer than 50 bp were included for further analysis, and the smallest variant relative to any of the eight MHC references was reported. The sequences of the calls so-defined were annotated with RepeatMasker (v.4.1.2)^91^. Variants were grouped by starting position on their closest MHC reference sequence and, in the case of insertions, repeat content was averaged.

#### Annotation

We applied Immuannot (see above) to all retrieved MHC loci for the identification and annotation of protein-coding HLA-DRB genes (*HLA*-*DRB1*, *HLA*-*DRB3*, *HLA*-*DRB4* and *HLA*-*DRB5*). Subsequently, a custom RepeatMasker (v.4.1.2)^91^ library was constructed containing the exonic sequences of HLA-DRB pseudogenes (*HLA*-*DRB2*: ENSG00000227442.1, *HLA*-*DRB6*: ENSG00000229391.8, *HLA*-*DRB7*: ENSG00000227099.1, *HLA*-*DRB8*: ENSG00000233697.2, and *HLA*-*DRB9*: ENSG00000196301.3) and RCCX genes and pseudogenes (*C4*: ENSG00000244731.10, *CYP21A2*: ENSG00000231852.9, *CYP21A1P*: ENSG00000204338.9, *STK19*/*RP1*: ENSG00000204344.16, *STK19B*/*STK19P*/*RP2*: ENSG00000250535.1, *TNXB*: ENSG00000168477.21 and *TNXA*: ENSG00000248290.1). Canonical exonic sequences were sourced from the Ensembl genome browser^108^ (release 111). The exons of HLA-DRB or RCCX genes and pseudogenes within individual haplotype MHC regions were annotated using this custom library. Repetitive elements were identified using RepeatMasker (v.4.1.2) with the Dfam library (v.3.4)^92^. We utilized SAMtools (v.1.15.1)^93^ and MUSCLE (v.3.8.31)^95^ for sequence retrieval and alignment, respectively, followed by manual annotation to analyse recombination events associated with DR subregion haplotypes and within the RCCX modules (Supplementary Methods). Novel *C4*-coding variants were identified through comparison with Ensembl *C4A* and *C4B* protein variant tables, as well as an additional database of variants obtained from 95 human MHC haplotypes^33^.

### Complex structural polymorphisms

#### CSV detection

CSVs were identified with a development version of PAV (methods are available^124^). In brief, the method identifies candidate variant anchors and scores variants between them. A directed acyclic graph is constructed with alignment records as nodes and variants connecting them as edges, which is solved in *O*(*N* + *E*) time with the Bellman–Ford algorithm^125^. Variants on the optimal path were accepted into the callset. CSVs intersecting centromeric repeats were eliminated. CSVs were merged into a non-redundant callset with SV-Pop by 50% reciprocal overlap and 80% sequence identity (SV-Pop merge parameter ‘nr::ro(0.5):match(0.8)’).

#### SMN analysis

We evaluated complexity and copy number of SMN genes by extracting with FASTA the desired region (chr. 5: 70300000–72100000) from assemblies reported in this study along with previously published assemblies^1,126^ (Supplementary Methods). Among these, we identified 101 fully assembled haplotypes. We followed this by aligning exon sequences for multicopy genes (*SMN1/2*, *SERF1A/B*, *NAIP* and *GTF2H2/C*) to each assembled haplotype. To assign a specific SMN copy to each haplotype, we extracted FASTA sequence from SMN exon regions for each haplotype and concatenated them into a single sequence (Supplementary Methods). We then constructed a multiple sequence alignment and calculated the distance among all haplotypes. We set the orangutan sequence as an outgroup and split all human haplotypes into two groups representing *SMN1* and *SMN2* gene copies where the SMN1 copy is the one closer to the outgroup. We utilized Illumina short-read data from the 1kGP for the same individuals, and processed it with Parascopy (v.1.16.0)^127^ and SMNCopyNumberCaller (v.1.1.2)^128^ to independently obtain *SMN1/2* copy numbers. Illumina-based and assembly-based copy number predictions matched perfectly across all 31 examined individuals.

### Centromeres

#### Centromere identification and annotation

To identify the centromeric regions within each Verkko and hifiasm (ultra-long) genome assembly, we first aligned the whole-genome assemblies to the T2T-CHM13 (v.2.0) reference genome^4^ using minimap2 (v.2.24)^78^ with the following parameters: -ax asm20 --secondary=no -s 25000 -K 15 G --eqx --cs. We filtered the alignments to only those contigs that traversed each human centromere, from the p to the q arm, using BEDtools (v.2.29.0)^129^ intersect. Then, we ran dna-brnn (v.0.1)^130^ on each centromeric contig to identify regions containing α-satellite sequences, as indicated by a ‘2’. Once we identified the regions containing α-satellite sequences, we ran RepeatMasker (v.4.1.0)^91^ to identify all repeat elements and their organization within the centromeric region. We also ran HumAS-HMMER (https://github.com/fedorrik/HumAS-HMMER_for_AnVIL) with the AS-HORs-hmmer3.0-170921.hmm model, which was trained on GRCh38 as previously described^58^, to determine the α-satellite HOR sequence composition and organization. We used the resulting RepeatMasker and HumAS-AMMER stv_row.bed files to visualize the organization of the α-satellite HOR arrays with R (v.1.1.383)^131^ and the ggplot2 package^132^.

#### Validation of centromeric regions

We validated the assembly of each centromeric region by first aligning native PacBio HiFi and ONT data from the same genome to each relevant whole-genome assembly using pbmm2 (v.1.1.0; for PacBio HiFi data; https://github.com/PacificBiosciences/pbmm2) or minimap2 (v.2.28)^78^ (for ONT data). We then assessed the alignments for uniform read depth across the centromeric regions via IGV^112^ and NucFreq^73^. Centromeres that were found to have a collapse in sequence, false duplication of sequence and/or misjoin were flagged and removed from our analyses.

#### Estimation of α-satellite HOR array length

To estimate the length of the α-satellite HOR arrays for each human centromere, we first ran HumAS-HMMER (https://github.com/fedorrik/HumAS-HMMER_for_AnVIL) on the centromeric regions using the hmmer-run.sh script and the AS-HORs-hmmer3.0-170921.hmm hidden Markov model. Then, we used the stv_row.bed file to calculate the length of the α-satellite HOR arrays by taking the minimum and maximum coordinate of the ‘live’ α-satellite HOR arrays, marked by an ‘L’, and plotting their lengths with GraphPad Prism (v9). We note that live or ‘active’ α-satellite HOR arrays are those that belong to an array that associates with the kinetochore in several individuals^58,133^. By contrast, ‘dead’ or ‘inactive’ α-satellite HORs (denoted with a ‘d’ in the HumAS-HMMER BED file) are those that have not been found to be associated with the kinetochore and are usually more divergent in sequence than the live or active arrays.

#### Pairwise sequence identity heatmaps

To generate pairwise sequence identity heatmaps of each centromeric region, we ran StainedGlass (v.6.7.0)^134^ with the following parameters: window = 5,000, mm_f = 30,000 and mm_s = 1,000. We normalized the colour scale across the StainedGlass plots by binning the percent sequence identities equally and recolouring the data points according to the binning.

#### CpG methylation analysis

To determine the CpG methylation status of each centromere, we aligned ONT reads of more than 30 kb in length from the same source genome to the relevant whole-genome assembly via minimap2 (v.2.28)^78^ and then assessed the CpG methylation status of the centromeric regions with Epi2me modbam2bed (https://github.com/epi2me-labs/modbam2bed; v.0.10.0) and the following parameters: -e -m 5mC --cpg. We converted the resulting BED file to a bigWig using the bedGraphToBigWig tool (https://www.encodeproject.org/software/bedgraphtobigwig/) and then visualized the file in IGV. To determine the length of the hypomethylated region (termed CDR^58,60^) in each centromere, we used CDR-Finder^135^. This tool first binned the assembly into 5-kb windows, computed the median CpG methylation frequency within windows containing α-satellite (as determined by RepeatMasker (v.4.1.0)^91^), selected bins that have a lower CpG methylation frequency than the median frequency in the region, merged consecutive bins into a larger bin, filtered for merged bins that are more than 50 kb and reported the location of these bins.

### Reporting summary

Further information on research design is available in the Nature Portfolio Reporting Summary linked to this article.

## Online content

Any methods, additional references, Nature Portfolio reporting summaries, source data, extended data, supplementary information, acknowledgements, peer review information; details of author contributions and competing interests; and statements of data and code availability are available at 10.1038/s41586-025-09140-6.

## Supplementary information

Supplementary Information Supplementary Text including Supplementary Results and Methods, Supplementary Figs 1–80 and Supplementary References – see Contents page for details.

Reporting Summary

Supplementary Tables Supplementary Tables 1–61.

## Data Availability

All data produced by the HGSVC and analysed as part of this study are available under the following accessions (see Supplementary Tables 2–4, 6–8 and 23 for details): PRJEB58376, PRJEB75216, PRJEB77558, PRJEB75190, PRJNA698480, PRJEB75739, PRJEB36100, PRJNA988114, PRJNA339722, PRJEB41778 and ERP159775 for PacBio HiFi and ONT LRS data; PRJEB39750 and PRJEB12849 for Strand-seq; PRJNA339722, PRJEB41077, PRJEB58376 and PRJEB77842 for Bionano Genomics; PRJEB39684, PRJEB75193 and PRJEB58376 for Hi-C; PRJEB75191 for PacBio Iso-Seq; PRJEB75192 and PRJEB58376 for RNA-seq; PRJEB76276 for phased genome assemblies generated by Verkko; and PRJEB83624 for phased genome assemblies generated by hifiasm. Released resources, including simple and complex variant calls, genome graphs, genotyping results (genome-wide and targeted), and annotations for centromeres, MEIs and SDs can be found in the IGSR release directory hosted publicly via HTTP and/or FTP (https://ftp.1000genomes.ebi.ac.uk/vol1/ftp/data_collections/HGSVC3/release) and on the Globus end point ‘EMBL-EBI Public Data’ in the directory ‘/1000g/ftp/data_collections/HGSVC3/release’.

## Code availability

All software, scripts and workflows used in this project that have not been formally published are publicly available via a central GitHub repository (https://github.com/hgsvc/phase3-main-pub; section ‘Software’) and on Zenodo^136^ (10.5281/zenodo.14546729).
