# Author Correction: Complex genetic variation in nearly complete human genomes

**DOI:** 10.1038/s41586-025-09547-1

**Published:** 2025-08-26

**Authors:** Glennis A. Logsdon, Peter Ebert, Peter A. Audano, Mark Loftus, David Porubsky, Jana Ebler, Feyza Yilmaz, Pille Hallast, Timofey Prodanov, DongAhn Yoo, Carolyn A. Paisie, William T. Harvey, Xuefang Zhao, Gianni V. Martino, Mir Henglin, Katherine M. Munson, Keon Rabbani, Chen-Shan Chin, Bida Gu, Hufsah Ashraf, Stephan Scholz, Olanrewaju Austine-Orimoloye, Parithi Balachandran, Marc Jan Bonder, Haoyu Cheng, Zechen Chong, Jonathan Crabtree, Mark Gerstein, Lisbeth A. Guethlein, Patrick Hasenfeld, Glenn Hickey, Kendra Hoekzema, Sarah E. Hunt, Matthew Jensen, Yunzhe Jiang, Sergey Koren, Youngjun Kwon, Chong Li, Heng Li, Jiaqi Li, Paul J. Norman, Keisuke K. Oshima, Benedict Paten, Adam M. Phillippy, Nicholas R. Pollock, Tobias Rausch, Mikko Rautiainen, Yuwei Song, Arda Söylev, Arvis Sulovari, Likhitha Surapaneni, Vasiliki Tsapalou, Weichen Zhou, Ying Zhou, Qihui Zhu, Michael C. Zody, Ryan E. Mills, Scott E. Devine, Xinghua Shi, Michael E. Talkowski, Mark J. P. Chaisson, Alexander T. Dilthey, Miriam K. Konkel, Jan O. Korbel, Charles Lee, Christine R. Beck, Evan E. Eichler, Tobias Marschall

**Affiliations:** 1 Department of Genome Sciences, University of Washington School of Medicine, Seattle, WA USA; 2 Department of Genetics, Epigenetics Institute, Perelman School of Medicine, University of Pennsylvania, Philadelphia, PA USA; 3 Core Unit Bioinformatics, Medical Faculty and University Hospital Düsseldorf, Heinrich Heine University, Düsseldorf, Germany; 4 Center for Digital Medicine, Heinrich Heine University, Düsseldorf, Germany; 5 The Jackson Laboratory for Genomic Medicine, Farmington, CT USA; 6 Department of Genetics and Biochemistry, Clemson University, Clemson, SC USA; 7 Center for Human Genetics, Clemson University, Greenwood, SC USA; 8 Institute for Medical Biometry and Bioinformatics, Medical Faculty and University Hospital Düsseldorf, Heinrich Heine University, Düsseldorf, Germany; 9 Program in Medical and Population Genetics and Stanley Center for Psychiatric Research, Broad Institute of MIT and Harvard, Cambridge, MA USA; 10 Center for Genomic Medicine, Massachusetts General Hospital, Boston, MA USA; 11 Department of Neurology, Massachusetts General Hospital and Harvard Medical School, Boston, MA USA; 12 Medical University of South Carolina, College of Graduate Studies, Charleston, SC USA; 13 Department of Quantitative and Computational Biology, University of Southern California, Los Angeles, CA USA; 14 Pathos AI Inc., Chicago, IL USA; 15 Institute of Medical Microbiology and Hospital Hygiene, Medical Faculty, Heinrich Heine University, Düsseldorf, Germany; 16 European Molecular Biology Laboratory, Wellcome Genome Campus, European Bioinformatics Institute, Cambridge, UK; 17 Department of Genetics, University of Groningen, University Medical Center Groningen, Groningen, The Netherlands; 18 Oncode Institute, Utrecht, The Netherlands; 19 Division of Computational Genomics and Systems Genetics, German Cancer Research Center, Heidelberg, Germany; 20 Department of Biomedical Informatics and Data Science, Yale School of Medicine, New Haven, CT USA; 21 Department of Biomedical Informatics and Data Science, Heersink School of Medicine, University of Alabama, Birmingham, AL USA; 22 Institute for Genome Sciences, University of Maryland School of Medicine, Baltimore, MD USA; 23 Department of Molecular Biophysics and Biochemistry, Yale University, New Haven, CT USA; 24 Program in Computational Biology and Bioinformatics, Yale University, New Haven, CT USA; 25 Department of Structural Biology, School of Medicine, Stanford University, Stanford, CA USA; 26 Genome Biology Unit, European Molecular Biology Laboratory (EMBL), Heidelberg, Germany; 27 UC Santa Cruz Genomics Institute, University of California, Santa Cruz, CA USA; 28 Genome Informatics Section, Center for Genomics and Data Science Research, National Human Genome Research Institute, National Institutes of Health, Bethesda, MD USA; 29 Department of Computer and Information Sciences, College of Science and Technology, Temple University, Philadelphia, PA USA; 30 Institute for Genomics and Evolutionary Medicine, Temple University, Philadelphia, PA USA; 31 Department of Data Science, Dana-Farber Cancer Institute, Boston, MA USA; 32 Department of Biomedical Informatics, Harvard Medical School, Boston, MA USA; 33 Department of Biomedical Informatics, University of Colorado School of Medicine, Aurora, CO USA; 34 Department of Immunology and Microbiology, University of Colorado School of Medicine, Aurora, CO USA; 35 Institute for Molecular Medicine Finland (FIMM), University of Helsinki, Helsinki, Finland; 36 Department of Computational Medicine and Bioinformatics, University of Michigan, Ann Arbor, MI USA; 37 Stanford Health Care, Palo Alto, CA USA; 38 New York Genome Center, New York, NY USA; 39 The University of Connecticut Health Center, Farmington, CT USA; 40 Howard Hughes Medical Institute, University of Washington, Seattle, WA USA; 41 Present Address: The Jackson Laboratory for Genomic Medicine, Farmington, CT USA

**Keywords:** Genomics, Genome informatics, Immunogenetics, Structural variation

Correction to: *Nature* 10.1038/s41586-025-09140-6 Published online 23 July 2025

In the version of the article initially published, the *x*-axis label for Fig. 4f was “Position (Mb)” and has now been corrected to “Position (kb)” in the HTML and PDF versions of the article.

