# Supplementary material for: Complex genetic variation in nearly complete human genomes
Source: Nature. 2025 Jul 23;644(8076):430–41. doi: 10.1038/s41586-025-09140-6 (PMC12350169; doi:10.1038/s41586-025-09140-6)
Supplement: Supplementary file 2 — Reporting Summary [file 41586_2025_9140_MOESM2_ESM.pdf]

Corresponding author(s): Tobias Marschall, Evan E. Eichler, Christine R. Beck, Charles Lee, Jan O. Korbel, Miriam K. Konkel

Last updated by author(s): Apr 18, 2025

Reporting Summary

Nature Portfolio wishes to improve the reproducibility of the work that we publish. This form provides structure for consistency and transparency in reporting. For further information on Nature Portfolio policies, see our Editorial Policies and the Editorial Policy Checklist.

Statistics

For all statistical analyses, confirm that the following items are present in the figure legend, table legend, main text, or Methods section.

n/a	Confirmed	
☐	☒	The exact sample size (*n*) for each experimental group/condition, given as a discrete number and unit of measurement
☐	☒	A statement on whether measurements were taken from distinct samples or whether the same sample was measured repeatedly
☐	☒	The statistical test(s) used AND whether they are one- or two-sided<br>*Only common tests should be described solely by name; describe more complex techniques in the Methods section.*
☒	☐	A description of all covariates tested
☐	☒	A description of any assumptions or corrections, such as tests of normality and adjustment for multiple comparisons
☐	☒	A full description of the statistical parameters including central tendency (e.g. means) or other basic estimates (e.g. regression coefficient) AND variation (e.g. standard deviation) or associated estimates of uncertainty (e.g. confidence intervals)
☐	☒	For null hypothesis testing, the test statistic (e.g. F, t, r) with confidence intervals, effect sizes, degrees of freedom and P value noted<br>*Give P values as exact values whenever suitable.*
☒	☐	For Bayesian analysis, information on the choice of priors and Markov chain Monte Carlo settings
☒	☐	For hierarchical and complex designs, identification of the appropriate level for tests and full reporting of outcomes
☒	☐	Estimates of effect sizes (e.g. Cohen's *d*, Pearson's *r*), indicating how they were calculated

Our web collection on statistics for biologists contains articles on many of the points above.

Software and code

Policy information about availability of computer code

Data collection	Pacific Biosciences (PacBio) high-fidelity (HiFi) long-read sequencing data was collected using SMRT Link v10.1, v12.0, and v13.0 (software version 10.1.0.119549), and Oxford Nanopore Technologies (ONT) long-read sequencing data was collected using PromethION software (v21.02.17 - 23.04.5). BioNano Genomics optical mapping data was collected via Saphyr 2nd generation instruments (Part #60325) using Instrument Control Software (ICS) v4.9.19316.1, and PacBio isoform-sequencing (Iso-Seq) data was collected with SMRT Link v12.0 and v13.0.
Data analysis	Custom software developed for this study include L1ME-AID (https://github.com/Markloftus/L1ME-AID) and MELT-LRA (github.com/Scott-Devine/MELT-LRA).<br><br>Custom scripts and pipelines developed for this study include those for sample selection (github.com/tobiasrausch/kmerdbg and github.com/asulovar/HGSVC3_sample_selection); Verkko genome assembly [github.com/core-unit-bioinformatics/workflow-smk-genome-hybrid-assembly (prototype branch)]; assembly evaluation [github.com/core-unit-bioinformatics/workflow-smk-assembly-evaluation (prototype branch)]; project-specific code for assembly-related evaluations, supplementary tables, and plots (github.com/core-unit-bioinformatics/project-run-hgsvc-assemblies); PanGenie genotyping and reference panel construction (github.com/eblerjana/hgsvc3); MEI, MHC, Iso-Seq, and SMN analysis (github.com/Markloftus/HGSVC3 and github.com/Markloftus/L1ME-AID); MHC annotation (github.com/DiltheyLab/MHC-annotation); and segmental duplication analysis (SDA2: https://github.com/ChaissonLab/SegDupAnnotation2).<br><br>All other software used in this study are publicly available and include Verkko (v1.4.1), hifiasm (v0.19.6), Graphasing (v0.3.1-alpha), MBG (v1.0.15 and v1.0.16), GraphAligner (v1.0.17 and v1.0.18), and MashMap (v3.0.6 and v3.1.3), Foreign Contamination Screening (FCS) (v0.4.0), minimap2 (v2.24, v2.26, and v2.28), SAMtools (v1.15.1 and v1.17), ISOGG (v15.73), CDR-Finder, modbam2bed (v0.10.0), bedGraphToBigWig, IGV, HMMER (v.3.3.2dev), TRF (v4.1.0), NAHRwhals, ArbiGent, ASHLEYS, MosaiCatcher(v2), Immuannot (MHC reference version: IPD-IMGT/HLA-V3.55.0), L1ME-AID (v1.0.0-beta), SNPrelate R package157 (v1.26.0), Factoextra (v1.0.7), NucFreq (NucFreq version "bd080aa" (from fork

NucFreqTwo / branch “split-two-phases”), Flagger (v0.3.3), Meryl (v1.0), Winnowmap2 (v2.03), DeepVariant (v1.6.0), bcftools (v1.17), Merqury (v1.0), compleasm (v0.2.5), OrthoDB (v10), mashmap (v3.1.3), PAV (v2.4.1), pbmm2 (v1.1.0, v1.5.0, and v1.12.0), LRA (v1.3.7.2), DipCall (v0.3), SVIM-asm (v1.0.3), PBSV (v2.9.0), Sniffles (v2.0.7), Delly (v1.1.6), cuteSV (v2.0.3), DeBreak (v1.0.2), SVIM (v2.0.0), DeepVariant (v1.5.0), DeepVariant executed through PEPPER-Margin-DeepVariant (vr0.8), Clair3 (v1.0.4), SV-Pop (v3.4.4), BCFtools (v1.16 and v1.17), BEDtools (v2.29.0 , v2.30.0, and v2.31.1), SciPy (v1.11.4), RepeatMasker (v4.1.0, v4.1.2, and v4.1.6), Biopython (v1.82), SEDEF (v1.1), Windowmasker (v2.2.22), seqtk (v1.3), vamos (v1.3.2), VCFtools (v.0.1.16), BEAST (v.1.10.4), RAxML (v.8.2.10), Tree-Annotator (v.1.10.4), FigTree software (v.1.4.4), Trimmomatic (v0.39), STAR (v2.7.10b), Cufflinks (v2.2.1), Lima (v2.1.0), isoseq3 (v3.8.2), SQANTI3 (v5.1.2), MUSCLE (v3.8.425), DESeq2 (v1.38.3), FAN-C (v0.9.26b2), Minigraph-Cactus (v2.7.2), PanGenie (v3.1.0), BLASTN (v2.14.1), rustybam (v0.1.33, 10.5281/zenodo.8106233), R (v1.1.383), R package ape (v5.7-1), R package phangorn (v2.11.1), Parascopy (v1.16.0), SMNCopyNumberCaller (v1.1.2), pgr-tk (v0.5.1), dna-brnn (v0.1), Graphpad Prism (v9), StainedGlass (v6.7.0), and Snakemake (v7.19.1).

For manuscripts utilizing custom algorithms or software that are central to the research but not yet described in published literature, software must be made available to editors and reviewers. We strongly encourage code deposition in a community repository (e.g. GitHub). See the Nature Portfolio guidelines for submitting code & software for further information.

Data

Policy information about availability of data

All manuscripts must include a data availability statement. This statement should provide the following information, where applicable:

- Accession codes, unique identifiers, or web links for publicly available datasets
- A description of any restrictions on data availability
- For clinical datasets or third party data, please ensure that the statement adheres to our policy

All data produced by the HGSVC and analyzed as part of this study are available under the following accessions: PacBio HiFi and ONT long reads: PRJEB58376, PRJEB75216, PRJEB77558, PRJEB75190, PRJNA698480, RJEB75739, PRJEB36100, PRJNA988114, PRJNA339722, PRJEB41778, ERP159775; Strand-seq: PRJEB39750, PRJEB12849; Bionano Genomics: PRJNA339722, PRJEB41077, PRJEB58376, PRJEB77842; HiC: PRJEB39684, PRJEB75193, PRJEB58376; PacBio Iso-Seq: PRJEB75191; RNA-seq: PRJEB75192, PRJEB58376. Released resources including simple and complex variant calls, graph genomes, genotyping results (genome-wide and targeted), and annotations for centromeres, mobile element insertions, and segmental duplications can be found in the IGSR release directory hosted publicly via HTTP and FTP (https://ftp.1000genomes.ebi.ac.uk/vol1/ftp/data_collections/HGSVC3/release) and on the Globus endpoint "EMBL-EBI Public Data" in directory "/1000g/ftp/data_collections/HGSVC3/working".

Research involving human participants, their data, or biological material

Policy information about studies with human participants or human data. See also policy information about sex, gender (identity/presentation), and sexual orientation and race, ethnicity and racism.

Reporting on sex and gender	We sequenced 65 human samples in this study, including 30 males (46,XY) and 35 females (46,XX).
Reporting on race, ethnicity, or other socially relevant groupings	N/A
Population characteristics	The samples included in the study represent 26 human populations, as defined by the 1000 Genomes Project.
Recruitment	Samples included in this study were of either African (n=30), Admixed American (n=9), European (n=8), East Asian (n=10), or South Asian (n=8) descent.
Ethics oversight	The lymphoblastoid cell lines and genomic DNA for each sample are available from the Coriell Institute for Medical Research (https://www.coriell.org/) for research purposes and are covered by the appropriate ethics approvals by the Coriell Institute.

Note that full information on the approval of the study protocol must also be provided in the manuscript.

Field-specific reporting

Please select the one below that is the best fit for your research. If you are not sure, read the appropriate sections before making your selection.

☒ Life sciences ☐ Behavioural & social sciences ☐ Ecological, evolutionary & environmental sciences

For a reference copy of the document with all sections, see nature.com/documents/nr-reporting-summary-flat.pdf

Life sciences study design

All studies must disclose on these points even when the disclosure is negative.

Sample size	A total of 65 human samples were included in this study.
Data exclusions	No data was excluded.
Replication	N/A. All computational analyses can be replicated using the provided codes and pipelines.

Randomization	N/A. Samples were not assigned to groups.
Blinding	N/A. All experiments were done computationally and do not involve a human experimenter.

Reporting for specific materials, systems and methods

We require information from authors about some types of materials, experimental systems and methods used in many studies. Here, indicate whether each material, system or method listed is relevant to your study. If you are not sure if a list item applies to your research, read the appropriate section before selecting a response.

Materials & experimental systems

n/a	Involved in the study
☒	☐ Antibodies
☐	☒ Eukaryotic cell lines
☒	☐ Palaeontology and archaeology
☒	☐ Animals and other organisms
☒	☐ Clinical data
☒	☐ Dual use research of concern
☒	☐ Plants

Methods

n/a	Involved in the study
☒	☐ ChIP-seq
☒	☐ Flow cytometry
☒	☐ MRI-based neuroimaging

Eukaryotic cell lines

Policy information about cell lines and Sex and Gender in Research

Cell line source(s)	All cell lines were obtained from the Coriell Institute for Medical Research (https://www.coriell.org/) and used to generate sequencing data, including: HG00096, HG00171, HG00268, HG00358, HG00512, HG00513, HG00514, HG00731, HG00732, HG00733, HG00864, HG01114, HG01352, HG01457, HG01505, HG01573, HG01596, HG01890, HG02011, HG02018, HG02059, HG02106, HG02282, HG02492, HG02554, HG02587, HG02666, HG02769, HG02818, HG02953, HG03009, HG03065, HG03248, HG03371, HG03452, HG03456, HG03520, HG03683, HG03732, HG03807, HG04036, HG04217, NA12329, NA18534, NA18939, NA18989, NA19036, NA19129, NA19238, NA19239, NA19240, NA19317, NA19331, NA19347, NA19384, NA19434, NA19650, NA19705, NA19836, NA19983, NA20355, NA20509, NA20847, NA21487, and NA24385.
Authentication	We did not authenticate the cell lines.
Mycoplasma contamination	According to information provided by the Coriell Institute for Medical Research, all cell lines are free of bacterial, fungal or mycoplasma contamination.
Commonly misidentified lines (See ICLAC register)	No commonly misidentified lines were used.

Plants

Seed stocks	N/A
Novel plant genotypes	N/A
Authentication	N/A
